# Sox17 Regulates Insulin Secretion in the Normal and Pathologic Mouse β Cell

**DOI:** 10.1371/journal.pone.0104675

**Published:** 2014-08-21

**Authors:** Diva Jonatan, Jason R. Spence, Anna M. Method, Matthew Kofron, Katie Sinagoga, Leena Haataja, Peter Arvan, Gail H. Deutsch, James M. Wells

**Affiliations:** 1 Division of Developmental Biology, Cincinnati Children’s Hospital Medical Center, Cincinnati, OH, United States of America; 2 Division of Endocrinology, Cincinnati Children’s Hospital Medical Center, Cincinnati, OH, United States of America; 3 Department of Internal Medicine, Division of Gastroenterology, University of Michigan Medical School, Ann Arbor, MI, United States of America; 4 Department of Cell and Developmental Biology, University of Michigan Medical School, Ann Arbor, MI, United States of America; 5 Center for Organogenesis, University of Michigan Medical School, Ann Arbor, MI, United States of America; 6 Division of Metabolism, Endocrinology, and Diabetes, University of Michigan Medical School, Ann Arbor, MI, United States of America; 7 Seattle Children’s Hospital, Seattle, WA, United States of America; Mayo Clinic, United States of America

## Abstract

SOX17 is a key transcriptional regulator that can act by regulating other transcription factors including HNF1β and FOXA2, which are known to regulate postnatal β cell function. Given this, we investigated the role of SOX17 in the developing and postnatal pancreas and found a novel role for SOX17 in regulating insulin secretion. Deletion of the *Sox17* gene in the pancreas (Sox17-paLOF) had no observable impact on pancreas development. However, Sox17-paLOF mice had higher islet proinsulin protein content, abnormal trafficking of proinsulin, and dilated secretory organelles suggesting that Sox17-paLOF adult mice are prediabetic. Consistant with this, Sox17-paLOF mice were more susceptible to aged-related and high fat diet-induced hyperglycemia and diabetes. Overexpression of *Sox17* in mature β cells using Ins2-rtTA driver mice resulted in precocious secretion of proinsulin. Transcriptionally, SOX17 appears to broadly regulate secretory networks since a 24-hour pulse of SOX17 expression resulted in global transcriptional changes in factors that regulate hormone transport and secretion. Lastly, transient SOX17 overexpression was able to reverse the insulin secretory defects observed in MODY4 animals and restored euglycemia. Together, these data demonstrate a critical new role for SOX17 in regulating insulin trafficking and secretion and that modulation of Sox17-regulated pathways might be used therapeutically to improve cell function in the context of diabetes.

## Introduction

Secretion of insulin by pancreatic β cells in response to glucose is central for glucose homeostasis, and dysregulation of this process is a hallmark of the early stages of diabetes. In healthy β cells, proinsulin transits through the endoplasmic reticulum (ER), where it is processed and folded, to the Golgi apparatus, where hexamerization is believed to occur, and finally, it is packaged into immature secretory granules that gradually mature into the final insulin storage organelle [1]. Within immature secretory granules, endoproteases Pcsk1/3, Pcsk2, and carboxypeptidase E cleave proinsulin to become mature insulin and c-peptide [2]–[6]. Prediabetes, full-blown type 2 diabetes, and genetic forms of diabetes, such as neonatal diabetes and maturity onset diabetes in youth (MODY), all share pathological features including elevated proinsulin levels in the plasma, dilated ER, elevated fasting blood glucose levels, and impaired glucose tolerance [7]–[17]. However, abnormal proinsulin processing or trafficking is known to be one of the hallmarks of prediabetes. This is supported by studies of the Akita mouse and in some patients with neonatal diabetes mellitus (NDM) that arise from mutations in the *Insulin* gene, resulting in improper folding of the mutant protein [7], [18], [19]. Akita mice have a progressive deterioration of secretory organelle structure and function, which is thought to be the primary cause of diabetes in these mice. Taken together, these studies indicate that genetic or acquired defects in proinsulin processing and/or trafficking can alter the homeostatic balance in β cells, resulting in impaired β cell function and a failure to maintain glucose homeostasis. Indeed, the regulatory molecules controlling insulin granule synthesis and secretion are not fully understood and may be exploited therapeutically to prevent or delay the progression of diabetes.

SOX17 is an HMG box transcription factor and a key regulator in various developmental and disease contexts, including endoderm organ development [20]–[26], primitive hematopoietic stem cell development, vascular development, tumor angiogenesis, and colon cancer cell proliferation [27]–[30]. Part of the ability of SOX17 to function in such diverse contexts is through its interactions with a diverse array of transcriptional co-factors including β-catenin, TCF/LEF, and Smad transcription factors. During endoderm development, SOX17 acts, in part, as a transcriptional regulator of other important endodermal transcription factors, including HNF1β and FOXA2, which are also known regulators of postnatal β cell function [31], [32]. While other SOX proteins have been implicated in islet cell development and homeostasis [33]–[35], a role for SOX17 in the adult β cell has not been described.

Here, we use a combination of mouse genetics, metabolic functional assays, high-resolution quantitation of subcellular localization of proinsulin and insulin, and microarray experiments to identify a novel role for SOX17 in regulating insulin trafficking and secretion in adult β cells both in normal and diabetic contexts.

## Research Design and Methods

### Mice

All experiments were performed in accordance with the recommendations in the Guide for the Care and Use of Laboratory Animals of the National Institutes of Health. The experiments were approved by the Committee on the Ethics of Animal Experiments at Cincinnati Children’s Hospital Research Foundation (3D06043 “Studies of metabolic and digestive disease in mice”). All details of all animal procedures are described below. For terminal experiments, animals were euthanized with a lethal amount of isoflurane inhalant, followed by a secondary method, cervical dislocation, and throughout experiments all efforts were made to minimize suffering. All mice used in these studies; *Pdx1-Cre*, *Pdx1-tTA*, *Sox17^fl^*, *Sox17^GFP^*, *Ins2-rtTA*, *tetO-Sox17*; have been previously described and were housed at the Cincinnati Children’s Hospital Research Foundation mouse facility [24], [25], [27], [36]–[39]. All mice were originally maintained on an outbred background and were then backcrossed to C57BL/6 background for at least 3 generations. Mature adult mouse group (12–24 weeks of age) was used in our analysis. For loss-of-function experiments, twelve- to sixteen-week-old adult male and female mice were used. For gain-of-function experiments using Ins2-rtTA driver, sixteen-week-old adult male mice were used. In order to upregulate Sox17 expression in this gain-of-function system, doxycycline (Dox) was given in the food and water for 24 hours before analysis, unless otherwise stated. For gain-of-function experiments using Pdx1-tTA driver, sixteen- to twenty-week-old adult mice were used, unless otherwise noted. We regulated Sox17 transgene expression with doxycycline as previously described [25]. Briefly, to keep the Sox17 transgene off, Pdx1-tTA;tetO-Sox17 mice were maintained on Dox chow, which maintains repression of the transgene. We removed Dox chow to induce expression of the Sox17 transgene.

### High fat diet-induced obesity

Mice were fed with a high-fat butter diet (60% fat, 5.24 kcal/g; Research Diets, New Brunswick, NJ, product no. D12492). Body weight and 4 hours fasting glucose and insulin levels were measured every week. Glucose tolerance test was performed after 21 weeks in high fat diet and insulin tolerance test was done at week 26. After 39 weeks, the mice were euthanized and pancreas were collected and analyzed. Analysis of islet number, islet area, and total pancreas area was performed on tile-scan images with Bitplane Imaris software. Briefly, total pancreas area was determined by generating an isosurface from the tissue autofluorescence channel. Isosurfaces were created for islets using intensity threshold for PDX 1 staining first, then selecting for PDX1 positive surfaces that contained high median pixel intensity values for insulin. Joined islets were carefully observed to determine whether they were connected and continuous. Some surfaces were manually split if they appeared separated on closer analysis. Data for islet number and area were generated via the statistics tab in the surface analysis window.

### Immunofluorescence and confocal microscopy analysis

Tissues were prepared and stained as previously described [25]. Six mice per genotype were analyzed; 3 sections per pancreas, for a total of 3–6 islets analyzed per mouse. Images were acquired using confocal microscopy using Zeiss LSM 510 with 40× dry objective at Nyquist limit. For Z-stack analysis, we used 63× PlanApo oil NA 1.4 objective at Nyquist limit and Nikon A1R si with 100× Apo oil NA 1.49 objective at Nyquist limit (2× zoom, 512×512 scan area). Full Z-stack images were created and analyzed using Bitplane Imaris 7.2 software. See Table S1 in File S1for a list of primary and secondary antibodies used in these studies. For pancreas area and islet quantification, images were acquired with a 20X plan apo objective on a Nikon A1R laser scanning confocal utilizing the resonant scanner, motorized XY stage and tile scanning utility in ND acquisition. Images were acquired for PDX1, Insulin and tissue autofluorescence (405 nm excitation). Overlap between tiles was 15%. A 3 airy unit pinhole was utilized to take a 7.5 um thick optical section. Tiles were stitched automatically using the Nikon Elements software.

### Islet isolation and total pancreatic insulin content

Islets were isolated using standard collagenase digestion followed by purification through a Ficoll gradient and islet gravity sedimentation. For total pancreatic insulin content, ten islets were handpicked, washed with Hank’s buffer (Gibco #14175), and lysed in 10 mM Tris-EDTA, 1% Triton-X 100, pH 8.0. Insulin content was measured using Mouse Insulin ELISA kit (Crystal Chem #90080).

### Non-fasting and fasting glucose, proinsulin, and insulin assays

Glucose was measured using Freestyle Freedom Blood Glucose Monitoring System. For fasting glucose, mice were fasted over night or as indicated. For plasma proinsulin and insulin levels, blood samples were taken either using tail-vein or submandibular bleed. Blood samples were incubated at room temperature for 20 to 30 minutes, followed by centrifugation for 5 minutes at 1,000 rpm. Plasma samples were then taken and centrifuged for 10 minutes at 2,000 rpm to eliminate the platelets. Plasma samples were analyzed using mouse Insulin and Proinsulin ELISA kits (Alpco #80-PINMS-E01).

### Western blot analysis of proinsulin and insulin

Proteins (3 mg/lane by BCA protein assay) were separated on 4–12% NuPAGE Novex Bis-Tris gels (Invitrogen), electrotransferred to nitrocellulose (Bio-Rad), and immunoblotted with guinea pig anti-insulin (Linco/Millipore) and mouse anti-tubulin (Sigma). Horseradish peroxidase-conjugated secondary antibodies were from Jackson ImmunoResearch with proteins visualized by enhanced chemiluminescence (ECL, Millipore).

### Glucose tolerance test and insulin tolerance test

Glucose tolerance test was performed as previously described [40]. For insulin tolerance test, mice were fasted for 8–12 hours and intraperitoneally injected with recombinant human insulin (1 U/kg, Novo Nordisk, Novolin®R NDC 0169-1833-11). Blood glucose levels were measured at indicated time points.

### Real-time PCR and Microarray analysis

Mouse islets were isolated as described above, and total islet RNA was extracted using either RNeasy Micro Kit (Qiagen, cat. no. 74004) or PureLink RNA Mini Kit (Invitrogen, cat. no. 12183-018A). RNA samples were then reverse transcribed into cDNA using the SuperScript III First-Strand Synthesis System (Invitrogen). For Real-time PCR, QuantiTect SYBR Green (Qiagen) was used on BioRad CFX96. For microarray analysis, RNA was isolated from islets and used to create target DNA for hybridization to Affymetrix Mouse 1.0 Gene ST Arrays using standard procedures (Affymetrix, Santa Clara, CA). Independent biological triplicates were performed for each genotype. Affymetrix microarray Cel files were subjected to RMA normalization in GeneSpring 10.1. Probe sets were first filtered for those that are overexpressed or underexpressed and then subjected to statistical analysis for differential expression by 1.3 fold or more between controls and either Sox17-paLOF or Sox17-GOF islets with p<0.05 using the Students t-test. Log2 gene expression ratios were then subjected to hierarchical clustering using the standard correlation distance metric as implemented in GeneSpring. Heat map was created using GeneSpring. The differentially expressed genes were subjected to Gene Ontology functional enrichment analyses using the ToppCluster server [41]. See Table S4 in File S1 for a list of primers used in these studies.

### Electron microscopy analysis

Mouse pancreas was dissected and fixed in 3% glutaraldehyde and 0.175 M sodium cacodylate buffer, pH 7.4, at 4°C for one hour. The samples were then post fixed in 1% osmium tetroxide in 0.2 M sodium cacodylate buffer for 1 hour at 4°C, processed through a graded series of alcohols, infiltrated, and embedded in LX-112 resin. After polymerization at 60°C for three days, ultrathin sections (100 nm) were cut using a Leica EM UC7 microtome and counterstained in 2% aqueous uranyl acetate and Reynolds lead citrate. Images were taken with a transmission electron microscope (Hitachi H-6750) equipped with a digital camera (AMT 2k×2K tem CCD). For immuno-gold staining, pancreatic tissue was fixed in 4% paraformaldehyde, 0.1% glutaraldehyde in PBS (pH 7.4) for 48 hours at 4°C, embedded in gelatin capsules after infiltration with LR White Resin (EMS, Hatfield, PA) and sectioned on a ultra-microtome. The thin cryosections were incubated with mouse monoclonal anti-proinsulin antibody (1∶25, R&D Systems) for 2 hours at room temperature, washed and labeled with goat anti-mouse IgG-gold (gold diameter 15 nm).

### Analysis of insulin subcellular localization

Surfaces were created using pixel intensity thresholds to identify positively stained proinsulin in the respective organelles and structures from background fluorescence. Imaris software calculated volumes for these structures and organelles using standard algorithm to measure total volume and relative percent colocalization. Images acquired with Zeiss LSM 5.10 were always acquired at 512×512 by 13 optical sections on average. This standard image volume size was used to compare the Sox17 animals to controls. Nikon images were tile-scanned to image the entire islet, and were always acquired at 512×512 by 56 optical sections on average. Due to variable size of the islets, total volume analysis was not included using the Nikon images.

### Statistical analysis

All the data are expressed as mean ± SEM, and Student t-tests were used for statistical analysis.

## Results

### *Sox17* is not required for β cell development

During the formation of the embryonic endoderm, SOX17 regulates several key transcription factors and signaling pathways that are known to play a central role in pancreas development and adult β cell homeostasis. We therefore investigated whether SOX17 might have a functional role in pancreas development and adult pancreas homeostasis by Cre-mediated inactivation of the *Sox17* gene (*Sox17^fl^*) [24], [25]. We used a Pdx1-Cre line that expresses Cre recombinase in the developing pancreas starting at e8.5 and drives efficient recombination throughout the pancreas [39]. In some contexts, we combined the *Sox17^fl^* with a null allele of Sox17 (*Sox17-GFP*) [27] as done previously [25]. Animals with a pancreatic knockout of *Sox17* are referred to as Sox17-paLOF (Figure S1A). We previously observed ectopic pancreatic tissue in the extrahepatic biliary ducts of mice where Sox17 was deleted with Foxa3-Cre [25] as indicated by the ectopic pancreas tissue shown here (Figure S1I, L). However, *Sox17* deletion mediated by the Pdx1Cre line driver used in these study, which deletes later in development, did not result in ectopic pancreatic tissue in the extrahepatic bile ducts (Figure S1H–K. Alcian blue was injected to highlight the duodenum and bile duct). At 12 weeks of age, the pancreas of Sox17-paLOF (*Sox17fl/fl;Pdx1-Cre*) animals appeared normal and gross islet architecture was no different from control animals as observed by the distribution of insulin, glucagon, and somatostatin expressing cells (Figure S1C–D). Quantitative analysis of islet mRNAs from isolated islets demonstrated that loss of *Sox17* had no effect on *Insulin*, *Somatostatin*, *Glucagon*, and *Glut2*, nor were there any changes in islet transcription factors such as *Foxa2*, *Gata6* and *HNF1b* (data not shown), which are known Sox17 targets in Xenopus endoderm [21], [22], [42]. We confirmed *Sox17* deletion in islets isolated from 12–16 week old of Sox17-paLOF animals using quantitative PCR, observing a 70% reduction in *Sox17* mRNA (Figure S1B, p-value≤0.05). The remaining expression is likely due to the reported expression of *Sox17* in endothelial cells [28], [30], [43], [44], which are not targeted by Pdx1-Cre. However, to confirm Pdx1-Cre activity in the islets we bred the r26r-lacZ reporter line into Sox17-paLOF mice and observed robust expression of the betagalactosidease protein in cells expressing Cre protein (Figure S1E–F) but not in islets of control mice. This confirms the robust activity of the Pdx1-Cre line [39], and taken together with the 75% reduction of Sox17 mRNA in islets from Sox17-paLOF suggests that Cre-mediated deletion is efficient. There were no overt changes in ductal or exocrine compartments of the Sox17-paLOF mice (data not shown).

### Sox17-paLOF results in elevated proinsulin protein in the islets

**Figure 1:**
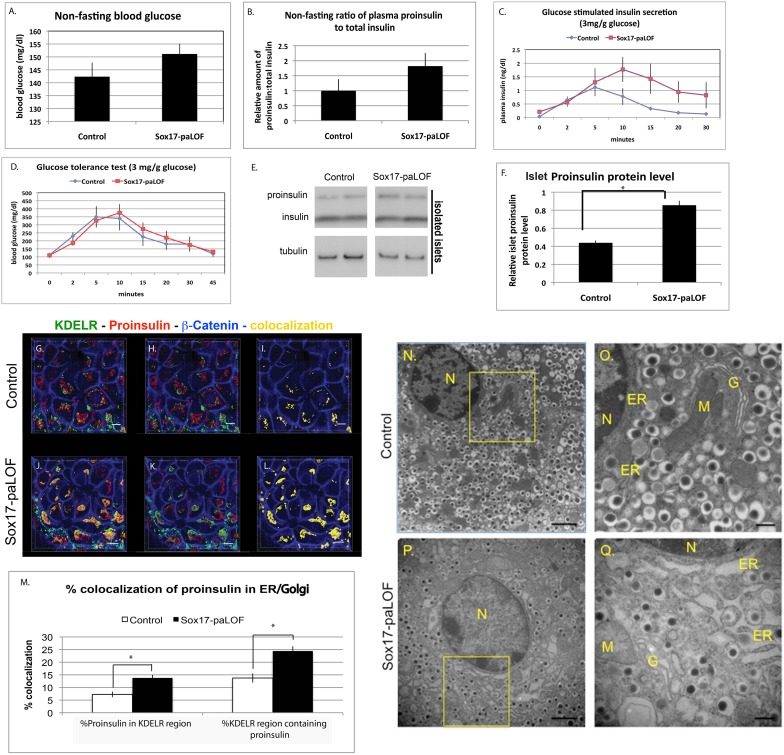
Sox17-paLOF results in proinsulin suβcellular localization and secretion changes and in the secretory organelles structural changes. **A, B)** There were no statistically significant differences in non-fasting blood glucose levels and total plasma insulin levels between control and Sox17-paLOF mice. Control mice were Sox17^fl/+^, Sox17^GFP/fl^; Pdx1Cre;Sox17^fl/+^, n = 19 for glucose measurement, n = 18 for plasma insulin measurement; Sox17-paLOF mice were Pdx1Cre;Sox17^GFP/fl^ and Pdx1Cre;Sox17^fl/fl^, n = 22 for glucose measurement, n = 20 for plasma insulin measurement. **C)** Glucose-stimulated insulin secretion showed higher trend of total plasma insulin level in Sox17-paLOF mice (Pdx1Cre;Sox17^GFP/fl^, n = 4) as compared to control mice (Sox17^fl/+^, n = 3). **D)** Glucose tolerance test showed similar glucose clearance (Control mice: Sox17^fl/+^, n = 4; Sox17-paLOF mice: Pdx1Cre;Sox17^GFP/fl^, n = 4). **E)** Analysis of isolated islets by western blot and subsequent quantification **F)** showed a significant increased in proinsulin protein in islets of Sox17-paLOF mice (asterisk shows p-value≤0.01). Control mice: Pdx1Cre;Sox17^fl/+^, n = 5. LOF mice: Pdx1Cre;Sox17^fl/fl^, n = 5. The western blot shows two representative examples. There was no difference in the islet insulin protein. **G–L)** Subcellular localization of proinsulin in mouse pancreatic β cells. Proinsulin levels were elevated in the secretory organelles as measured by proinsulin co-staining with the dynamic pre-Golgi and Golgi to the ER transport marker, KDELR. G and J showed staining of KDELR, Proinsulin, β-Catenin, and colocalization between KDELR and Proinsulin; H and K showed KDELR, Proinsulin, and β-Catenin only; I and L showed the colocalization pattern and β-catenin staining only. Proinsulin (red), KDELR (green), β-catenin staining of the cytoskeleton (blue), and colocalization between KDELR and Proinsulin is shown in yellow. Scale bar: 5 µm. **M)** Quantitation of percent ER and proinsulin colocalization using Bitplane Imaris software. The p-values (*) were ≤0.01 for Control (Sox17^fl/+^ and Sox17^GFP/fl^, n = 7–8) and Sox17-paLOF mice (Pdx1Cre;Sox17^GFP/fl^, n = 7). Between 6–10 islets were analyzed per mouse. **N–Q)** Analysis of β cells by transmission electron microscopy (TEM) indicated that the ER, Golgi (G), and mitochondria (M) were dilated in the Sox17-paLOF mice (Pdx1Cre;Sox17^fl/fl^, n = 3) relative to the Controls (Pdx1Cre;Sox17^fl/+^ (n = 3), Sox17^fl/+^, n = 2). N = nucleus. Black vesicles = insulin granules. Scale bar: 500 nm for N and P, 2 µm for O and Q.

We next investigated if Sox17 plays a role in the physiological function of adult β cells. At 12–16 weeks of age, Sox17-paLOF mice had normal non-fasting blood glucose and total plasma insulin levels by ELISA that detects all forms of insulin including proinsulin, mature insulin, and C-peptide (Figure 1A and data not shown). However, analysis of the ratio of proinsulin to total insulin levels showed a trend towards increased proinsulin (relative to insulin) in the plasma of Sox17-paLOF animals as compared to controls (Figure 1B). When challenged with an intraperitoneal (IP) bolus of glucose, the levels of total proinsulin+insulin in the plasma tended to be higher in Sox17-paLOF mice (Figure 1C). Interestingly, despite higher levels of total plasma insulin, Sox17-paLOF mutant mice did not exhibit any improvement in glucose tolerance (Figure 1D). Elevated levels of serum insulin without a corresponding reduction in blood glucose could be due to insulin insensitivity or accumulation of inactive proinsulin in the serum. Sox17-LOF animals had normal peripheral insulin sensitivity and normal levels of total insulin protein and mRNA in isolated islets (Figure S2A, B, C). To determine if there were elevated levels of unprocessed proinsulin, we analyzed protein extracts from isolated islets by western blot (Figure 1E). While there was no difference in the amount of mature insulin islet protein between genotypes, Sox17-paLOF islets contained significantly elevated proinsulin levels relative to control islets (Figure 1E and F). These data indicate that Sox17-paLOF mice have an increased islet proinsulin-to-insulin ratio resulting in elevated plasma and islet proinsulin levels, a feature that has been considered as an early indicator of β cell dysfunction associated with prediabetes.

### Loss of *Sox17* in the pancreas alters subcellular distribution of proinsulin and causes structural changes in secretory organelles

We investigated if loss of Sox17 resulted in altered levels of proinsulin processing enzymes or in altered proinsulin trafficking. There was no difference in the mRNA levels of the proinsulin processing proteins *Pcsk1*, *Pcsk2*, or *Cpe* in Sox17-paLOF islets (data not shown). However, we did observe changes in proinsulin trafficking by quantifying proinsulin subcellular distribution along the secretory pathway using the Imaris Software. Due to the subcellular dynamic of proinsulin trafficking, three different markers for secretory organelles were used, KDELR, ERGIC, and GM130. KDELR marks the dynamic transport between the pre-Golgi and Golgi to the ER [45]–[47]. Thus, quantifying proinsulin colocalization with KDELR marker represents the overall dynamic transport of proinsulin through these secretory organelles. In addition, ERGIC specifically marks the pre-Golgi compartment and GM130 marks the Golgi subcellular region.

While there was no difference in the accumulation of proinsulin in either the pre-Golgi (ERGIC) or Golgi (GM130) compartments across genotypes (Figure S3A–D), proinsulin appeared to accumulate in KDELR+ vessicles in Sox17-paLOF animals (Figure 1 G–M). KDELR is found both in the Golgi and ER and plays an important role in retrograde transport from Golgi to ER. Since proinsulin levels were normal in the pre-Golgi and Golgi, the increase of KDELR+/pro-insulin+ co-staining may represent an increase in proinsulin-containing vesicles that are recycling through the ER or an increase in improperly packabed insulin. There was no change in the pre-Golgi, Golgi, or ER volume area between genotypes (data not shown).

These findings suggest that abnormal proinsulin trafficking through the secretory organelles might be the underlying cause of altered proinsulin processing and secretion as observed in other contexts [7], [17], [48]. Proinsulin trafficking defects are also associated with distended and dilated secretory organelles that occur during the prediabetes phase in Akita mice [7], [14], [17]. Similarly, β cells of Sox17-paLOF mice had severely distended ER, pre-Golgi, and Golgi compared to *Pdx1Cre;Sox17^fl/+^* and *Sox17^fl/fl^* animals, analyzed by electron microscopy (EM) (Figure 1N–Q). These marked organelle distention were specific to the β cells; the alpha and delta cells had normal, well-organized secretory organelles (data not shown). There was a trend towards fewer insulin granules in the Sox17-paLOF mice, although this was not statistically significant because of variation between animals and sections. Taken together, these data suggested that Sox17-paLOF mice are prediabetic.

### Sox17-paLOF mice develop age-induced β cell dysfunction

**Figure 2:**
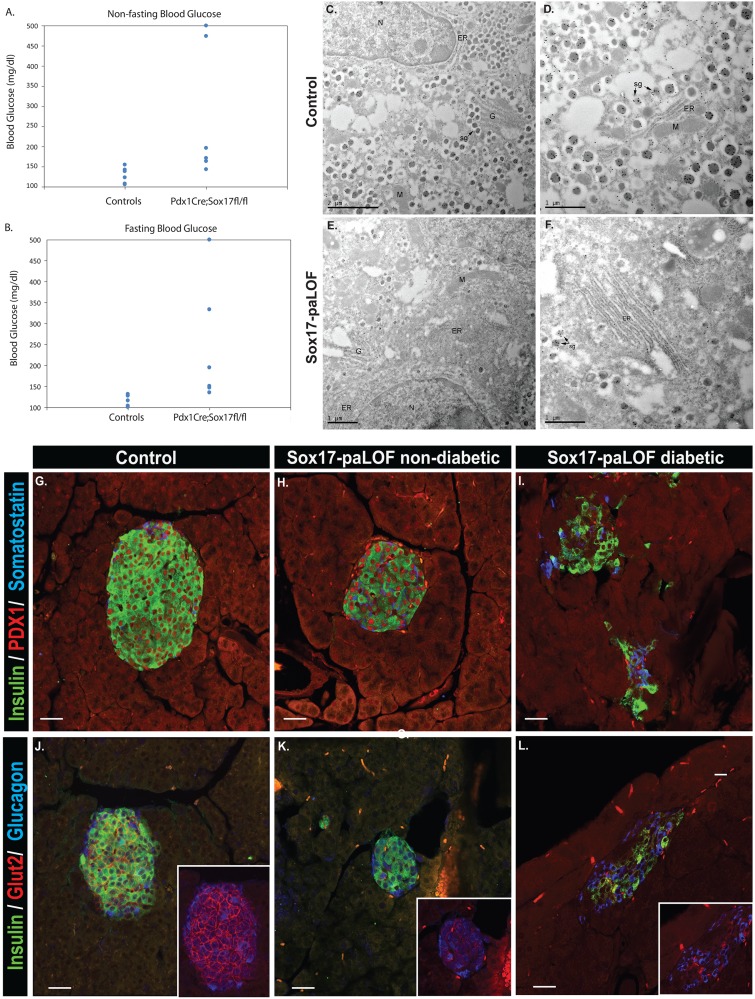
Sox17-paLOF mice become hyperglycemic and develop diabetes as they age. **A–B)** Non-fasting and fasting states of Sox17-paLOF and control mice at 1.5 years of age showing variation of blood glucose levels in the Sox17-paLOF group. **C–F)** Proinsulin immunogold analysis of β cells by TEM. **C–D)** Control mice contained proinsulin (black dots) labeling in the ER, Golgi, and secretory granules (sg). **E–F)** Sox17-paLOF β cells had many less well-formed secretory containing immunogold insulin granules, localized to the ER, dispersed ER, and secretory granules. N = nucleus, Black vesicles = insulin granules, G = Golgi, M = Mitochondria. Scale bar: 1 µm, except for C - scale bar: 2 µm. **H-M)** Insulin, Pdx1, Somatostatin, and Glucagon staining showed β cell mass reduction and disorganized islet architecture in diabetic Sox17-paLOF mice (Fasting glucose: >500 mg/dl, 2I, L), compared to non-diabetic Sox17-paLOF mice (Fasting glucose: 195 mg/dl, 2H, K) and control mice (Pdx1Cre;Sox17^fl/+^, fasting glucose: 128 mg/d, 2G, J). **J-L)** Glut2 protein levels was lost in diabetic Sox17-paLOF (L) compared to non-diabetic Sox17-paLOF (K) and control mice (J).

Previous studies have shown that an elevated proinsulin to insulin ratio within patients is an indicator of prediabetes for type 2 diabetes and a characteristic of β cell impairment in maturity onset diabetes [10]–[16]. To determine if loss of *Sox17* predisposes animals to diabetes progression as they get older, we analyzed these mice at 1.5 years of age. All aged Sox17-paLOF mice had some degree of defectiveness in reagrds to glucose homeostasis, with some mice being mildy hyperglycemic to others being fully diabetic during both non-fasting and fasting periods (Figure 2A, B). In the diabetic mice we observed reduced β cell numbers, shown by insulin and PDX1 immunostainings (Figure 2G–I) when compared with the non-diabetic Sox17-paLOF and control mice. The islets in these mice also had disrupted islet architecture with glucagon and somatostatin positive cells no longer restricted to the islet periphery (Figure 2G–L). In addition, all Sox17-paLOF mice had severely reduced levels of Glut2 (Figure 2J–L). This is in line with previous studies showing reduction in Glut2 expression level in various type 2 diabetes animal models, including Zucker Diabetic Fatty (ZDF) rats, diabetic Wistar Kyoto rats, and db/db mice [49]–.

To determine specific proinsulin subcellular trafficking in these older mice, we analyzed immunodetectable proinsulin epitope using TEM. In the control mice, the proinsulin was localized to the Golgi, ER, and secretory granules. In the Sox17-paLOF mice, the ER was very disorganized/distended and we observed many less well-formed secretory granules containing immunogold-labeled proinsulin compared to the controls (Figure 2C, D compare to 2E, F). In these Sox17-paLOF mice, proinsulin was observed in the ER, golgi, and secretory granules. Taken together, our findings showed that Sox17-paLOF mice were prediabetic and they were prone to reach diabetic phase as they aged.

### Sox17-paLOF mice are prone to high fat diet-induced β cell dysfunction

**Figure 3:**
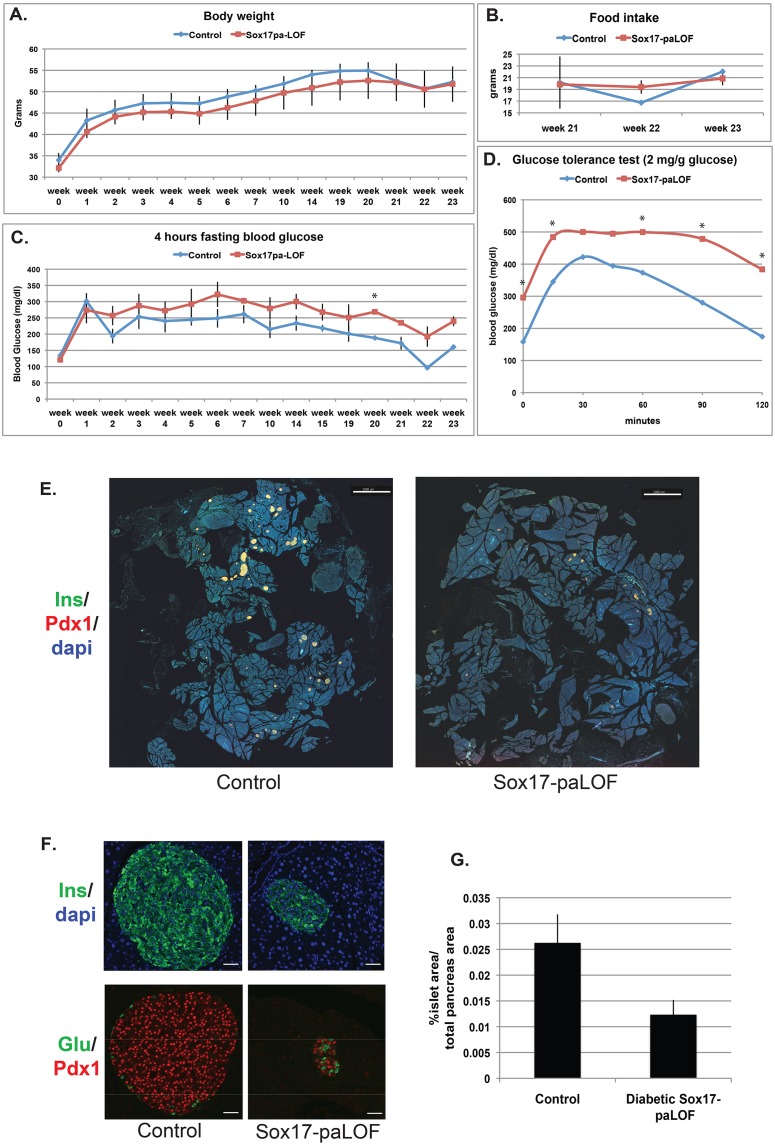
Sox17-paLOF mice develop high fat diet-induced β cell dysfunction and lose β cell mass. **A)** Over the course of 23 weeks on a high fat diet, there was no difference in weight gain between Control (Pdx1Cre;Sox17^fl/+^, n = 3) and Sox17-paLOF (Pdx1Cre;Sox17^fl/fl^, n = 4–6) littermates. **B)** Food intake measurements showed no difference between genotypes. **C)** Analysis of fasting blood glucose levels. Sox17-paLOF had higher fasting glucose levels than control mice. Asterisk show p-value≤0.01. **D)** Glucose tolerance test showed impaired glucose clearance in the Sox17-paLOF mice 21 weeks after high fat diet administration. The asterisks indicate p-value≤0.05. **E)** Impact of Sox17-paLOF on islets. Pancreatic sections (n = 2 mice per genotype, 6 whole pancreatic sections analyzed per mouse) from each animal were stained with insulin, Pdx1, and Dapi and indicated significant reduction in islet size in Sox17-paLOF mice. Scale bar: 2000 µm. **F)** Islet organization is disrupted in Sox17-paLOF mice. Insulin, Pdx1, and glucagon staining showed loss of β cell mass and a prevalence of alpha cells in the islet core of Sox17-paLOF mice. Scale bar: 30,000 µm **G)** Quantification of loss of islet mass in Sox17-paLOF mice. The islet and total pancreas surface areas were calculated using Imaris Software. (n = 2 mice per genotype, 6 whole pancreatic sections analyzed per mouse).

Given the predisposition of Sox17-paLOF mice to age related diabetes, we investigated if loss of *Sox17* predisposes animals to high-fat diet-induced diabetes. Six month old Sox17-paLOF males and their *Pdx1Cre;Sox17^fl/+^* littermates were maintained on a high-fat diet and monitored for 7 months. We observed that mice of all genotypes had a similar weight gain in response to a high-fat diet (Figure 3A), similar amount of food intake and insulin peripheral sensitivity between genotypes as assayed by an insulin tolerance test (Figure 3B and Figure S4). However, Sox17-paLOF mice on high-fat diet had impaired fasting glucose (Figure 3C) and were unable to restore normoglycemia in response to a glucose challenge as quickly as control animals (Figure 3D).

However, as in the case with aged-induced diabetic mice, the levels of dysfunction were also varied in the Sox17-paLOF obese mice, with some mice being mildy hyperglycemic to others being fully diabetic. We investigated if this range was due to variability of β cell function or due to reduced β cell number. We analyzed whole pancreatic sections taken at different levels throughout the pancreas and quantified the number and size of islets in mice 39 weeks after being exposed to high fat diet (Figure 3E, G). All control mice had numerous large islets associated with the increased metabolic stress caused by obesity [52]. In contrast, Sox17-paLOF obese mice that were diabetic had profoundly reduced islet size (Figure 3F and quantified in G). These animals also had disrupted islet architecture with many alpha cells found in the core. We found no evidence of co-expression of alpha and beta cell markers such as insulin, PDX1 and glucagon (Figure 3F). The size and islet architecture of mildly hyperglycemic Sox17-LOF animals were comparable to control mice (data not shown). Taken together, these data suggest that Sox17-paLOF mice have β cell dysfunction, are prediabetic, and are prone to high fat diet-induced diabetes.

### SOX17 overexpression for 24 hours promotes proinsulin trafficking and secretion *in vivo*

**Figure 4:**
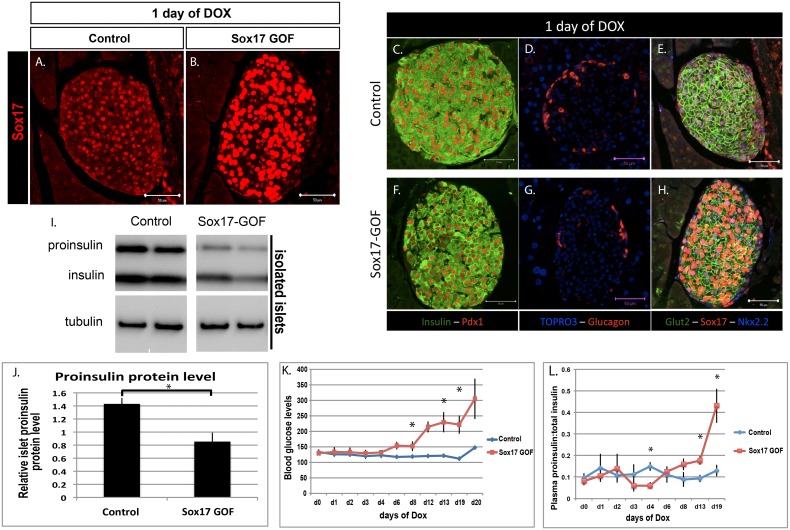
Sox17 overexpression for 24 hours is sufficient to alter proinsulin:total insulin protein ratio and proinsulin secretion *in vivo*, followed by accumulation of proinsulin in the plasma, leading to diabetes after prolonged exposure of Sox17. **A and B)** Analysis of Sox17 protein expression in control (TetO-Sox17) and Sox17 Gain-of-Function (GOF) (Ins-rtTA;TetO-Sox17) mice fed with doxycycline chow (dox) for 24 hours. **C–H)** Immunofluorescence analysis of islet proteins 24 hours after Sox17 overexpression. There were no overt changes in Insulin, Pdx1, Glucagon, and Glut2 levels and no alteration in islet architecture was observed in Sox17-GOF mice. **I)** Western blot of insulin and proinsulin from pancreatic islets of control (n = 3) and Sox17-GOF (n = 5) islets. **J)** Quantification of insulin and proinsulin from (I) showed significant reduction in proinsulin protein in islets (p-value≤0.01). **K)** Non-fasting blood glucose levels over 20 days of Sox17 overexpression showed an increase in blood glucose levels (n = 6 for Controls and n = 5 for Sox17-GOF islets, asterisks show p-value≤0.05). **L)** Ratio of plasma proinsulin to total insulin levels in control (n = 3) and Sox17-GOF (n = 3) mice. Sox17 overexpression caused a 4-fold increase of proinsulin levels in the plasma (asterisks show p-value≤0.05). Scale bar: 50 µm.

The above experiments suggest that Sox17-paLOF mice have proinsulin trafficking and secretory defects that predispose them to type 2 diabetes. However, they do not specifically address whether Sox17 regulates the cellular machinery responsible for proinsulin trafficking and secretion. We therefore utilized a tetracycline-inducible approach to investigate the immediate impact of a pulse of Sox17 expression on the insulin secretory pathway. Sox17 gain-of-function animals (Sox17-GOF) were generated using an *Ins2-rtTA* mouse line [36], [37] and a line in which Sox17 expression is regulated by the tetracycline transactivator (*tetO-Sox17*) [24], [25] (Figure S5A). Administering doxycycline to mice for 24 hours resulted in Sox17 overexpression in mature β cells in the islets of 16 week-old mice (Figure 4A and B). There were no changes in islet architecture as determined by insulin and glucagon stainings (Figure 4C–H). However, SOX17 overexpression was sufficient to cause a significant reduction in proinsulin protein level in the islets with no significant difference in mature insulin level (Figure 4I, J and Figure S5B), in contrast to the accumulation of proinsulin that we observed in the islets of Sox17-paLOF mice (Figure 1E–F). Analysis of plasma indicated that SOX17 overexpression was stimulating secretion of proinsulin and over time resulted in a four-fold increase in the ratio of proinsulin levels, resulting in these animals becoming hyperglycemic (Figure 4K and L).

**Figure 5:**
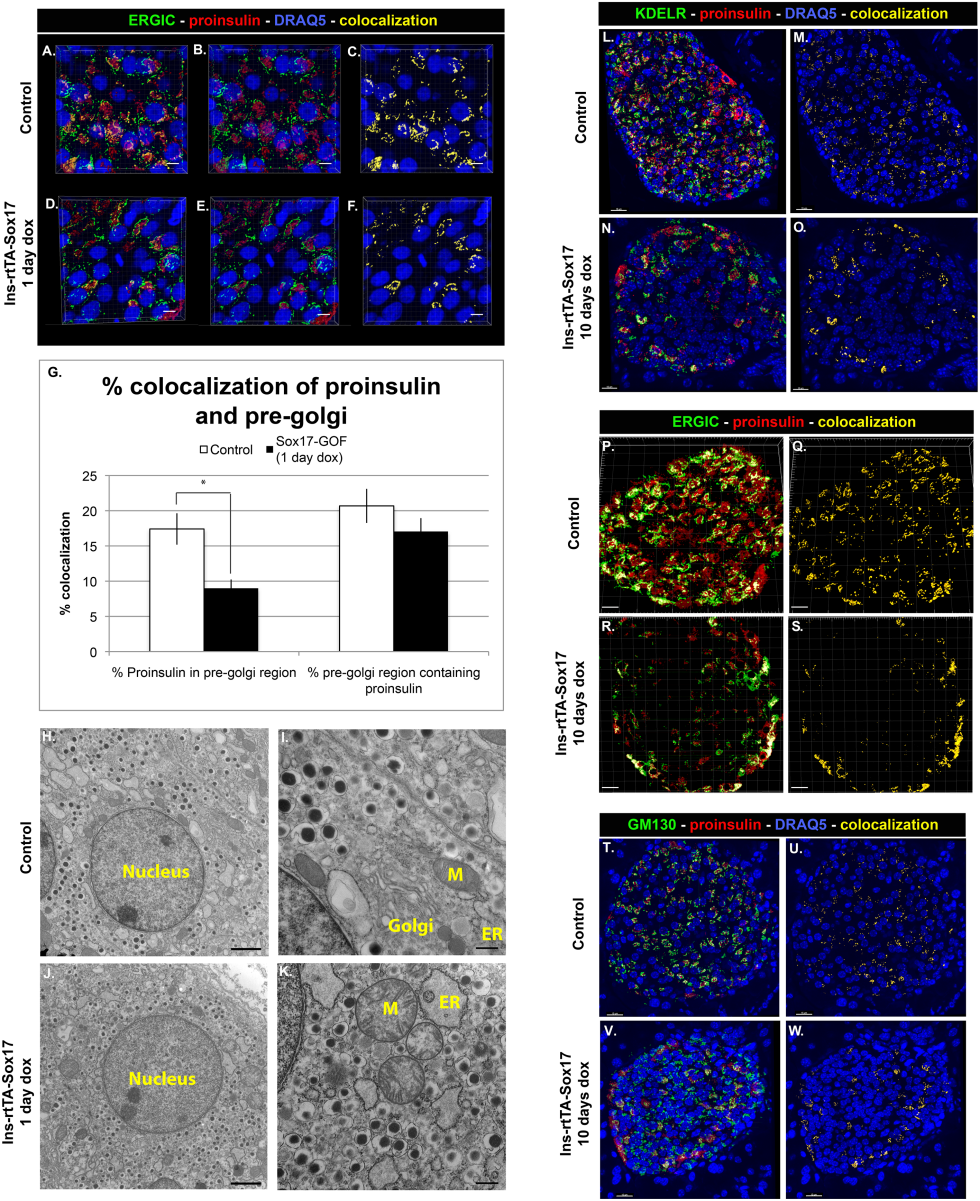
Sox17 overexpression alters trafficking through the secretory pathway. **A–F)** Proinsulin levels were reduced in the pre-Golgi (ERGIC) of Sox17 GOF mice (n = 3) relative to controls (n = 3, 4–6 islet sections per mouse analyzed). Proinsulin (red), ERGIC (green), DRAQ5/nuclei (blue). Sacle bar: 5 µm. **G)** Quantification of percent pre-Golgi and proinsulin colocalization using Bitplane Imaris software (asterisk shows p-value≤0.01). **H–K)** EM image analysis showed dilated morphology of ER and mitochondria in the Sox17-paGOF islet sections. ER = endoplasmic reticulum, G = Golgi, M = mitochondria. Scale bar: 500 nm for H and J, 2 µm for I and K. **L–W)** Proinsulin was reduced in all compartments of the secretory pathway following 20 days of Sox17 expression. Proinsulin levels in the ER and Golgi (KDELR), Pre-Golgi (ERGIC), and Golgi (GM130) were analyzed in control (n = 4) and Sox17-GOF (n = 3) mice. Scale bar: 15 µm, except for N - scale bar: 10 µm.

Analysis of proinsulin trafficking indicated that within 24 hours of SOX17 overexpression in β cells, proinsulin was transiting through the pre-Golgi faster than normal as indicated by a decrease in proinsulin within the pre-Golgi (Figure 5A–F, quantified in 5G). Consistent with this, total pre-Golgi area was also found to be decreased in these mice (Figure S6O), indicating that the impairment in the pre-Golgi compartment was impacting the proinsulin trafficking. There was no difference in proinsulin within the Golgi or ER regions (Figure S6M, N). The effects of SOX17 expression for 24 hours appeared to be limited to proinsulin secretion and processing since we did not observe any significant changes in mRNA levels of insulin or insulin processing enzymes (data not shown) and gross islet architecture was unchanged in these animals (Figure 4C–H). However, after 10 days of Sox17 overexpression, we observed broad loss of proinsulin throughout the secretory system (Figure 5L–W) correlated with hyperglycemia and an increase of proinsulin in the plasma, suggesting that Sox17 overexpression was causing constitutive secretion of proinsulin. We also observed distended secretory organelles suggestive of a general secretory defect in SOX17 overexpressing animals (Figure 5H–K). To determine if this was permanent, we repressed the *tetO-Sox17* transgene by removal of Doxycycline. Within 25 days, animals had normal blood glucose levels and islet architecture (Figure S5C–P), suggesting that the effect of Sox17 on insulin was reversible and that it was not due to a change in cell fate. Together, these data demonstrate that SOX17 expression stimulates the insulin secretory pathway within 24 hours and that continued expression promoted precocious proinsulin secretion and hyperglycemia.

### SOX17 transcriptome: SOX17 regulates pathways involved in insulin transport and secretion

**Figure 6:**
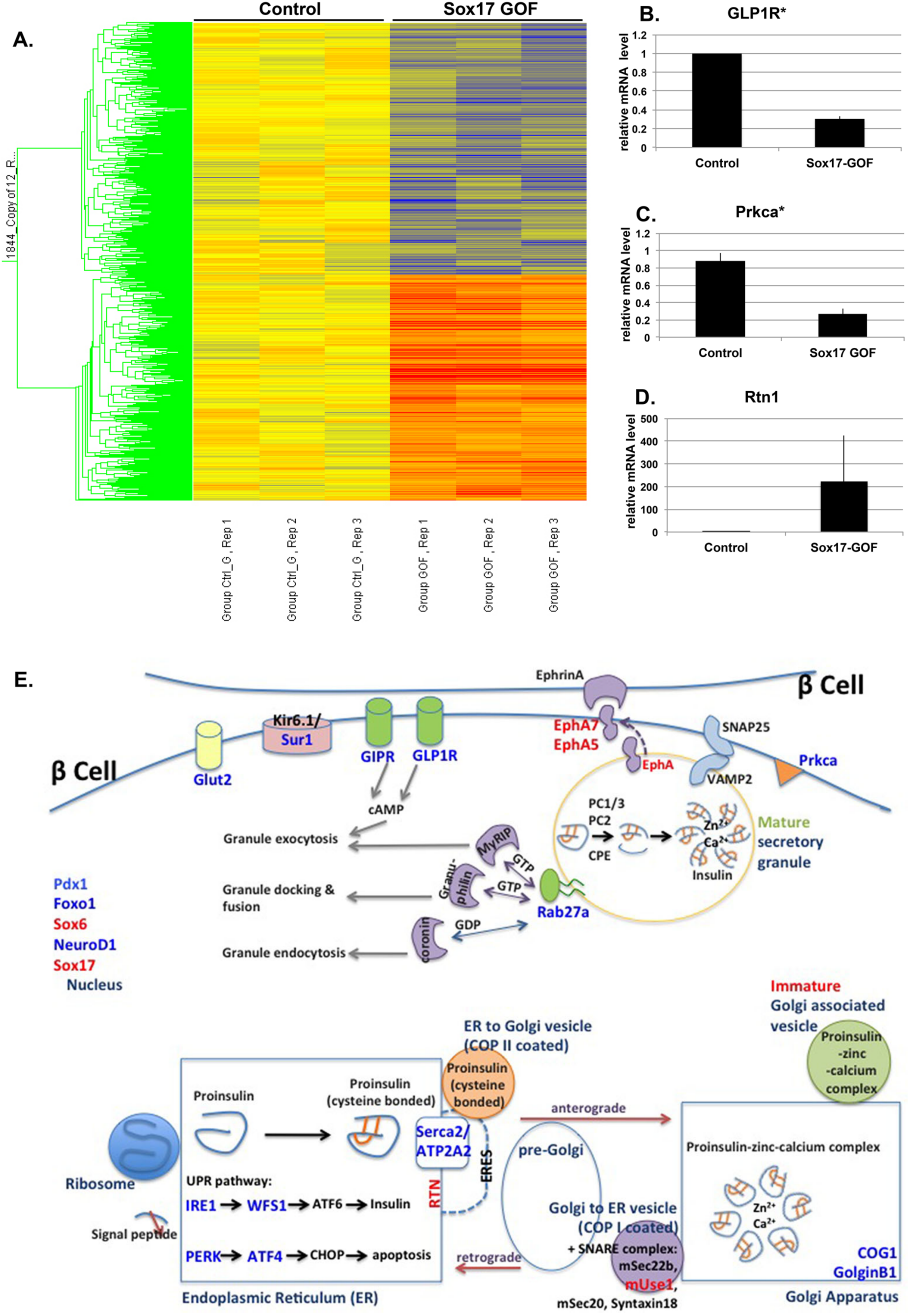
Sox17 regulates pathways involved in insulin transport and secretion. **A)** Analysis of transcriptional changes in islets after 24 hours of Sox17 overexpression by microarray. The heat map highlights the Sox17-regulated transcripts (upregulated genes in red, downregulated genes in blue, n = 3 per genotype). **B–D)** Quantitative-RTPCR validation of transcripts that were up- or down-regulated in response to Sox17 including Glp-1 receptor (Glp1R), protein kinase C isoform a (Prkca), Reticulon 1 (Rtn1), all of which are known regulators of secretory pathway. **E)** Schematic representation of Sox17-regulated factors and their known involvement in the insulin processing and secretory pathways. Genes that were upregulated by Sox17 expression are shown in red, and those that were down regulated are shown in blue.

In order to identify the molecular basis by which Sox17 regulates the secretory pathway in β cells, we performed microarray analysis on isolated islets following a 24-hour pulse of Sox17 overexpression (Figure 6A). We chose to analyze a 24-hour pulse of Sox17 since it was sufficient to stimulate the insulin secretory pathway without causing changes in islet architecture. We found 1844 transcripts in islets that were altered by 1.3 fold or more in response to Sox17 expression: 972 genes were reduced and 872 genes were elevated (an abbreviated list is shown in Table S2 in File S1). Transcripts were classified into gene ontology categories that represent significantly altered cell-biological processes using ToppCluster software (p<0.05) with no correction (Table S3 in File S1). The most highly regulated biological processes in response to SOX17 overexpression included peptide/insulin transport, localization and secretion, consistent with our proposed role of SOX17 in regulating insulin trafficking and secretion. Other enriched processes include cell development and cell morphogenesis. We confirmed the SOX17-responsiveness of 24 genes by qRTPCR (Figure 6B–D and Figures S7 and S8), suggesting that the microarray data set is highly robust. Several Sox17 regulated genes are schematically depicted in Figure 6E and were grouped into their known roles in insulin trafficking and secretion. Given the rapid transcriptional response of secretory networks to SOX17 expression, and the corresponding changes in insulin trafficking and secretion, these data suggest that SOX17 is a major and direct regulator of the insulin secretory pathway. All of the microarray data can be found on the GEO database and the accession number is GSE59928.

### Transgenic SOX17 expression restores some β cell functions in a MODY4 background

**Figure 7:**
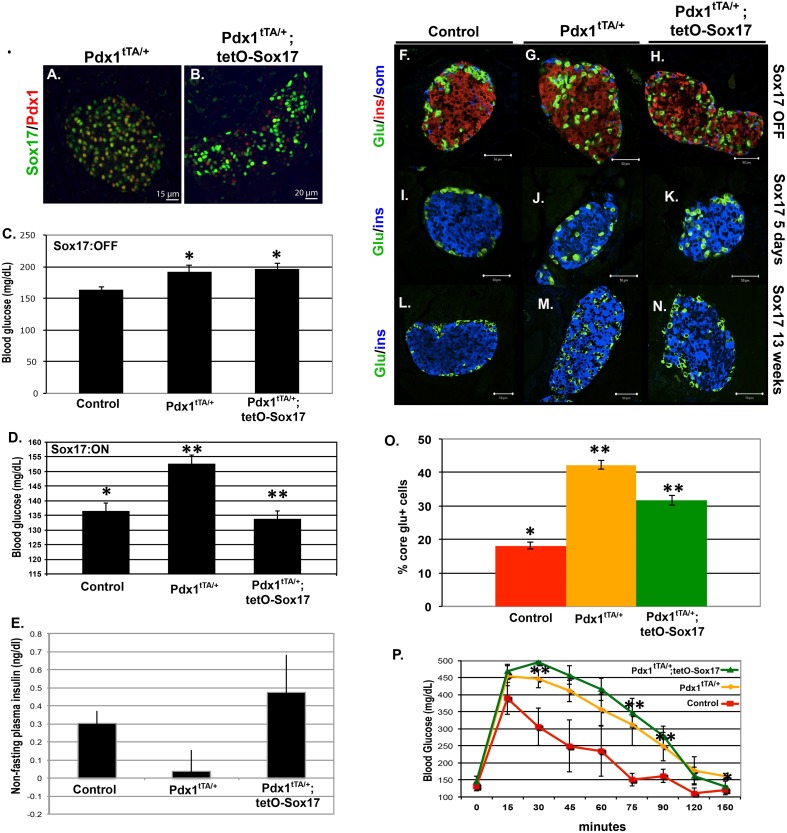
Sox17 expression in β cells improves glycemic control and islet architecture but not glucose clearance in MODY4 (Pdx1t^TA/+^) animals. **A, B)** Tetracycline-regulated expression of Sox17 in Pdx1t^TA/+^ animals. Sox17 levels in Pdx1 haploinsufficient animals (Pdx1t^TA/+^) were compared to animals with transgenic Sox17 overexpression driven by the Pdx1^tTA/+^ allele (Pdx1^tTA/+^;TetO-Sox17). Activation of the TetO-Sox17 transgene by removal of doxycycline resulted in a 2–3 fold increase in Sox17 protein over Pdx1^tTA/+^ control animals. Scale bar: 15 µm for A and 20 µm for B. **C, D)** Non-fasting blood glucose levels in control (TetO-Sox17), Pdx1^tTA/+^, and Pdx1t^TA/+^;TetO-Sox17 mice on doxycycline (Sox17 OFF, n = 3) and off doxycycline (Sox17 ON, n = 3) for 8 days. **E)** Non-fasting plasma insulin levels were low in Pdx1t^TA/+^ animals compared to controls. Sox17 expression restored serum insulin to control levels. **F–O)** Islet architecture was disrupted in Pdx1^tTA/+^ animals, with glucagon (+) cells in the core of the islet. The islet architecture was more normal appearing following 13 weeks of Sox17 expression in Pdx1t^TA/+^ animals. Scale bar: 50 µm. **P)** Sox17 did not restore the ability of Pdx1^tTA/+^ animals to respond to a glucose challenge (Pdx1-tTA;tetO-Sox17 mice – n = 3, off Dox; Pdx1-tTA – n = 2, on Dox and n = 1, off Dox; tetO-Sox17– n = 2, on Dox and n = 1, off Dox).

Perturbations in insulin secretion are a common feature of several genetic forms of diabetes. Given the ability of SOX17 to regulate the insulin secretory pathway, we investigated if SOX17 overexpression might improve β cell function in a diabetic context. In particular, we wanted to investigate if a modest increase in Sox17 levels might have therapeutic benefit. To do this, we chose a mouse model for maturity onset diabetes of the young (MODY4), which is caused by Pdx1 haploinsufficiency [53]–[57] and can be modeled using *Pdx1^tTA/+^* mice. Importantly, with this model we were able to use the tetracycline transactivator (tTA) in the *Pdx1^tTA/+^* allele to express Sox17 protein in a tetracycline-regulated manner (Figure 7A, B). We observed that *Pdx1^tTA/+^* mice exhibit previously reported MODY4 symptoms including hyperglycemia, reduced plasma insulin, impaired glucose tolerance, and aberrant localization of alpha cells to the core of the islet (Figure 7C–P). At the time of weaning, *Pdx1^tTA/+^* and *Pdx1^tTA/+^;tetO-Sox17* mice kept on doxycycline (no *Sox17* transgene expression) had elevated blood glucose levels relative to control animals (Figure 7C). However, removal of doxycycline after weaning and subsequent overexpression of SOX17 in the MODY4 background (*Pdx1^tTA/+^;tetO-Sox17* off dox) for 8 days was sufficient to restore glucose levels to normal (Figure 7D). In addition, glucose was also restored in these *Pdx1^tTA/+^;tetO-Sox17* mice after 13–17 weeks of SOX17 prolonged overexpression (data not shown, age of mice during time of analysis: 16–20 weeks old). This was correlated with restoration of normal plasma insulin levels (Figure 7E). Moreover, one of the pathologies observed in MODY4 mice is disruption of localization of glucagon positive alpha cells to the core of islets. We observed that the prolonged expression of SOX17 for 13–17 weeks after weaning (age of mice during time of analysis: 16–20 weeks old) resulted in near restoration of normal islet architecture with most alpha cells being localized to the periphery of the islets (Figure 7F–N, quantitated in O). To note, we observed that the blood glucose rescue was more effective early after SOX17 was being overexpressed, and that the islet architecture rescue was more effective after prolonged SOX17 overexpression. Previous studies by Johnson, et al. showed that the MODY4 phenotype coincided with increased in β cell apoptosis [55]; however, this MODY4 model did not display any significant changes in apoptosis or proliferation at this 13–17 weeks of overexpression time point (Figure S9). Lastly, MODY4 is commonly associated with an inability to respond to a glucose challenge. Despite restoration of normal resting glucose and insulin levels, SOX17 expression did not reverse the glucose intolerance of *Pdx1^tTA/+^* mice (Figure 7P). This suggests that SOX17 expression can rescue impaired fasting glucose but does not rescue impaired glucose tolerance.

## Discussion

Collectively, our results suggest a new role for the transcription factor Sox17 in regulating the mouse cell secretory pathway, a dynamic and highly regulated process. Pancreatic loss of Sox17 resulted in trafficking defects of proinsulin through the ER, dilated and distended secretory organelles, and a trend of increased secretion of proinsulin (Figure S10C), all of which are hallmarks of prediabetes [7]–[17]. Consistant with this, these mice went on to develop diabetes when placed on a high fat diet or when aged, as evidenced by loss of glucose regulation, severely reduced β cell mass and fewer secretory granules, as well as diminished Glut2 protein expression.

Overexpression of SOX17 in mature β cells resulted in rapid changes in insulin trafficking and over time caused a 4-fold increased in unprocessed proinsulin in the plasma (Figure S10B). We investigated if SOX17 could impact insulin secretory defects in MODY4 animals and found that a modest increase in SOX17 levels transiently normalized glucose and plasma insulin levels in diabetic MODY4 mice. Lastly, microarray data suggested a direct role for SOX17 in the regulation of proinsulin trafficking and secretion *in vivo* by regulating key genes involved in hormone transport, secretion, and cellular localization.

Several candidate genes, including SOX17, were investigated for a role in neonatal diabetes mellitus in humans [58], however no SOX17 mutation were associated with this form of diabetes. This is not surprising given the absolute requirement of Sox17 in a myriad of developmental processes outside of the β cell. However, it is possible that factors that act downstream of Sox17 might be association with prediabetes. Our investigation of Sox17 regulated transcripts clearly indicates a central role for this factor in regulating insulin secretory pathways, and it is possible that some of these might be deranged in prediabetes in humans. Of the 30 biological processes that are changed by Sox17 expression, 18 are involved in insulin/hormone/peptide trafficking and secretion. We schematically depicted several Sox17-regulated genes in Figure 5E and focused on their known role in insulin trafficking and secretion. For example, *Reticulon1* (*Rtn1*) (4-fold increase) is required for insulin trafficking and may explain the faster trafficking of proinsulin from the ER to Golgi. *Rtn1* is known as curvature-stabilizing protein that is localized in the ER exit sites (ERES), important to form and maintain a tubular ER morphology [59], which are known to affect the organization of the ER to Golgi transport system [60]. Overexpression of Reticulon in PC12 cells was also found to significantly enhance human growth hormone secretion [61]. It is known that Reticulon interacts with several SNARE proteins involved in vesicle exocytosis, including syntaxin13, 7, 1, and VAMP2 [59]–[62] and we found that SOX17 increases expression of SNARE proteins such as USE1, which could affect anterograde trafficking and overall transit through the ER and Golgi.

SOX17 regulated genes also control insulin secretion in response to fasting and feeding periods. These factors include the transmembrane tyrosine kinase receptors Ephrin (EPH) A5 and A7, which repress insulin secretion in low glucose [63]. Genes that were decreased in response to SOX17 expression included *Glp1r* and *Glut2*, both involved in nutrient-stimulated insulin secretion, as well as the small G-protein *Rab27a*, a member of the Rab GTPase protein family that is known to regulate insulin secretion at several levels depending on the downstream effectors being regulated [64], [65]. This suggested a role for SOX17 in regulating the secretion process. The hyperglycemia observed in the obese Sox17-paLOF mice in response to fasting and glucose challenge is consistent with this conclusion. SOX17 also down regulates *Prkca* (1.67 fold), which is known to translocate to the plasma membrane upon glucose stimulation and play a role in glucose-stimulated insulin secretion [66]–[68]. These changes in molecules that regulate basal and nutrient stimulated insulin secretion could explain the elevated glucose levels in Sox17-GOF mice over time.

Lastly, we observed changes in β cell transcription factors including *Pdx1*, *NeuroD1*, *Foxo1*, all reduced between 1.3 to 1.4 fold. While this is a relatively modest change in expression, these factors have central roles in β cell homeostasis and insulin secretion [69]; changes in the levels of these factors could have a significant combined impact. While we are highlighting a handful of transcripts that are changed in response to 24 hours of SOX17 overexpression, the known role of these genes is entirely consistent with the changes in insulin trafficking and secretion that we observe in the Sox17-GOF mice and indicate a new role for SOX17 as a regulator of insulin secretion in the adult β cell.

There were no changes in several known SOX17 target genes encoding the transcription factors HNF1β and FOXA2. This suggests that SOX17 may partner with different transcriptional co-factors in β cells to target a different subset of genes. While there were a lot of genes that were downregulated by SOX17 overexpression and knowing that there is no evidence of SOX17 acting as a repressor, we did observe an increase in the expression of PDX1 co-repressor, SOX6. Previous study in MIN6 cells showed that SOX6 decreased PDX1 stimulation through changes in chromatin structure on the insulin promoter [35]. It was suggested that SOX6 reduce glucose-stimulated insulin secretion in hyperinsulinemic obese mice by acting as corepressor of PDX1. These studies and our study further suggested that there is a complex β cell network of transcription factors that work together to govern insulin secretion process, and a change in one of the important nodes in the network can dramatically change the effects of the other transcription factor.

Given the requirement of SOX17 in endoderm and extrahepatobiliary cell fate specification, we expected that SOX17 may play a role in pancreatic development. It was curious that deletion of *Sox17* in the pancreas did not have a more pronounced effect on organ development. There are several SOX proteins expressed in the developing and adult murine pancreas, including SOX4, 5, 6, 9, 10, 11, 12, 13, and SOX15 [33]–[35]. Moreover, SOX17 is expressed at high levels in the human fetal pancreas and at lower levels in adult islets [70]. It is possible that other SOX proteins in the adult islets compensate for the loss of SOX17 in our Sox17-paLOF mice [28], [34], [70], [71]. For example, SOX4 is the most highly expressed in the islets; it is known to be important for endocrine differentiation, islet organization [33], and for mediating insulin secretion in response to glucose [72]. In addition, SOX9 is also known to regulate *Pdx1* expression and glucose-stimulated insulin release [73]. Taken together, these results suggest that other SOX proteins may compensate for the *Sox17* loss during development of the pancreas, but they do no compensate for the role of *Sox17* in regulating several important aspects of insulin secretion.

In summary, our findings support a model in which the transcription factor SOX17 controls multiple aspects of insulin trafficking in mature β cells through transcriptional regulation of genes involved in the maintenance of secretory machinery and insulin secretion. Our results suggested that absence of *Sox17* in adult pancreatic β cells results in mice with prediabetic symptoms including improper secretion of proinsulin, abnormal secretory organelles, and development of diabetes when aged and in response to a high fat diet. This model could provide a better insight into the causes of prediabetic phase, and will inform effort to help design a preventive therapeutical strategy that can be useful for human patients.

## Supporting Information

Figure S1 **Sox17 is not required for β cell development. A)** Schematic representation of the Sox17-paLOF mice. Pdx1-Cre, Sox17^fl^, and Sox17^GFP^ lines have been previously published [1]–[3]. **B)** Quantification of fold difference in Sox17 transcript showed significant reduction of Sox17 mRNA levels in Sox17-paLOF islets (asterisk (*) shows p-value≤0.05). Real Time RT-PCR samples were normalized to GAPDH mRNA. (Control mice: Pdx1-Cre;Sox17^fl/+^, n = 4; Sox17-paLOF mice: Pdx1Cre;Sox17^GFP/fl^, n = 3) **C–D)** Immunofluorescence using anti-insulin, -somatostatin, -and glucagon in control and Sox17-paLOF mice show no difference in islet architecture between control and Sox17-paLOF mice. Scale bar: 50 µm. **E–F)** Immunofluorescence using anti-cre and anti-beta-galactosidase in control and Sox17-paLOF mice containing a r26r-lacZ reporter allele demonstrating Cre expression and efficient recombination in islets. There is significant background staining with the beta galactosidase antibody in the exocrine compartment of the pancreas. Scale bar: 50 µm. **G–L)** Whole mount images of Pdx1Cre;Sox17^fl/fl^, Foxa3Cre;Sox17^fl/fl^ and control mice at e16.5. The duodenum of Pdx1Cre;Sox17^fl/fl^ and control animals was injected with alcian blue to provide contrast in the common bile duct. No ectopic pancreas was observed in Pdx1Cre;Sox17^fl/fl^ and control animals, in contrast to Foxa3Cre;Sox17^fl/fl^ mice that have ectopic pancreatic tissue (arrowheads) in the common duct as previously reported [2].
(JPG)

Figure S2 **Islet insulin levels and peripheral insulin sensitivity are unaffected in Sox17-paLOF mice. A, B)** Isolated islets were isolated from control and Sox17-paLOF mice and were analyzed for total insulin mRNA (Control mice: Sox17^fl/+^, n = 2, and Pdx1-Cre;Sox17^fl/+^, n = 1; Sox17-paLOF mice: Pdx1Cre;Sox17^GFP/fl^, n = 5) and protein (Control mice: Pdx1Cre;Sox17^fl/+^, n = 4; Sox17-paLOF mice: Pdx1Cre;Sox17^GFP/fl^, n = 3). **C)** Animals were tested for peripheral insulin sensitivity by injection of insulin as previously described [4]. There were no changes in insulin sensitivity in Sox17-paLOF mice (Control mice: Sox17^+/fl^, n = 2; Sox17-paLOF mice: Pdx1Cre;Sox17^GFP/fl^, n = 4).
(JPG)

Figure S3 **Percent colocalization between proinsulin and organelle markers, and their total regional areas. A, B)** Immunofluorescence analysis of proinsulin localization in the pre-Golgi (ERGIC) and Golgi (GM130). Scale bar: 5 µm. **C, D)** Quantification of A and B indicate that there were no differences found in the levels of colocalization between pre-Golgi and proinsulin, and between Golgi and proinsulin. Quantitation of proinsulin colocalization was performed using Bitplane Imaris software. Control: Sox17^fl/+^ and Sox17^GFP/fl^, n = 7 mice, Sox17-paLOF: Pdx1Cre;Sox17^GFP/fl^, n = 7. 6–10 islets were analyzed per mouse.
(JPG)

Figure S4 **Insulin tolerance test of obese control and Sox17-paLOF mice.** Obese animals (26 weeks after high fat diet administration) were fasted for 8–12 hours and intraperitoneally injected with recombinant human insulin (1 U/kg). Control mice: Pdx1Cre;Sox17^fl/+^, n = 3; Sox17-paLOF mice: Pdx1Cre;Sox17^fl/fl^, n = 4. Blood glucose levels were measured at the indicated time points.
(JPG)

Figure S5 **A tetracycline-regulated model for Sox17 overexpression. A)** Schematic representation of the Sox17-GOF mice. Ins-rtTA and TetO-Sox17 animals have been described previously [2], [5]–[7]. **B)** Insulin protein levels are not significantly changed after 24 hours of Sox17 overexpression. **C)** Hyperglycemia is induced by prolonged dox-inducible Sox17 overexpression, but reverts to normal within 25 days following doxycycline removal (Sox17 off). **D–P)** Analysis of Sox17, Insulin, Glucagon, Pdx1 and E-cadherin in control, Sox17 overexpressing (Doxycycline ON), and following removal of doxycycline for 25 days. Scale bar: 50 µm.
(JPG)

Figure S6 **Distribution of proinsulin in the Golgi and ER of Sox17-GOF mice. A–L)** Immunofluorescence analysis of proinsulin localization in the ER and Golgi (KDELR) and Golgi only (GM130) in control and Sox17-GOF mice. Scale bar: 5 µm. **M and N)** Quantification of proinsulin, KDELR, and GM130 staining found no change in the percent of proinsulin in the ER and Golgi **O)** Quantitation of total proinsulin and pre-Golgi area.
(JPG)

Figure S7 **Quantitative RT-PCR validation of down-regulated genes in response to 24 hours of Sox17 overexpression in β cells.** **A, B)** Insulin and Pdx1 mRNA were decreased, but this was not statistically significant. **C–P)** Glut2, Foxo1, Atf4, GLP1R, Hdac6, Prkca, Pkd1, Lpl, Defb1, Cpb2, Vilip-1, Insrr, Rab27a, Wfs were all significantly down regulated in response to 24 hours of Sox17 overexpression in β bells. Asterisk indicates p-value≤0.05. **Q)** Ppp1r1a was highly reduced in response to Sox17 overexpression, but this was not statistically significant.
(JPG)

Figure S8 **Quantitative RT-PCR validation of up-regulated genes in response to 24 hours of Sox17 overexpression in β cells.** **A)** Gsta4, **B)** Mobp, **C)** Lipf, **D)** Use1, and **E)** Rrn1 are examples of transcripts that were elevated in β cells in response to 24 hours of Sox17 overexpression. Asterisk indicates p-value≤0.05.
(JPG)

Figure S9 **Sox17 expression in MODY4 (Pdx1^+/tTA^) mice did not alter β cell proliferation or β cell death. A–D)** MODY4 (Pdx1^+/tTA^) mice had comparable levels of BrdU+ cells to both control (Wildtype or tetO-Sox17) and MODY4 (Pdx1^+/tTA^) mice expressing Sox17. N = 4 animals per genotype. **E–H)** MODY4 (Pdx1^+/tTA^) mice had comparable levels of activated caspase3+ cells to both control (Wildtype or tetO-Sox17) and MODY4 (Pdx1^+/tTA^) mice expressing Sox17. N = 4 animals per genotype. Scale bar: 50 µm.
(JPG)

Figure S10 **Schematic of β cells in different contexts. A)** Normal β cell schematic with normal mitochondria, ER, Pre-Golgi, and Golgi structures and secretory vesicles. Preproinsulin containing secretory vesicle is in red, proinsulin containing secretory vesicle is in blue, and insulin + C-peptide containing secretory vesicle is in green. Arrows show the anterograde (yellow arrow) and retrograde (orange arrow) movements of the vesicles. **B)** Sox17 overexpressing β cell schematic. The cell had less amount of proinsulin vesicles trafficking through pre-Golgi region. Over time, a 4-fold increased in unprocessed proinsulin is found in the plasma. **C)** Prediabetic Sox17-paLOF β cell schematic at 12 weeks old. The cell had dilated and distended secretory organelles, accumulated proinsulin, and a trend of increased secretion of proinsulin. **D)** Diabetic Sox17-paLOF β cell schematic at 1.5 years old. Some of the ER were dispersed and dilated and the cells had less insulin in the secretory organelles and granules. Overall, the mice had reduced β cell mass.
(PDF)

File S1 **Supporting tables and references.** Table S1, Primary and Secondary Antibodies. Table S2, Transcripts that were changed in Sox17-GOF islets. List of transcripts change by >1.55 fold in Sox17-GOF islets. Red highlighted genes were upregulated, blue highlighted genes were downregulated. Table S3, Gene ontology analysis of biological pathways and processes associated with SOX17 regulated transcripts. List of gene ontology of the biological process that are involved in the Sox17-misregulated genes cluster in the Sox17-GOF islets microarray by 1.30 fold change and above (upregulated genes cluster in red, downregulated genes cluster in blue). Table S4, PCR primers for microarray genes validation.
(DOCX)
