# Supplementary figures and images for: Sox17 Regulates Insulin Secretion in the Normal and Pathologic Mouse β Cell
Source: PLoS One. 2014 Aug 21;9(8):e104675. doi: 10.1371/journal.pone.0104675 (PMC4140688; doi:10.1371/journal.pone.0104675)

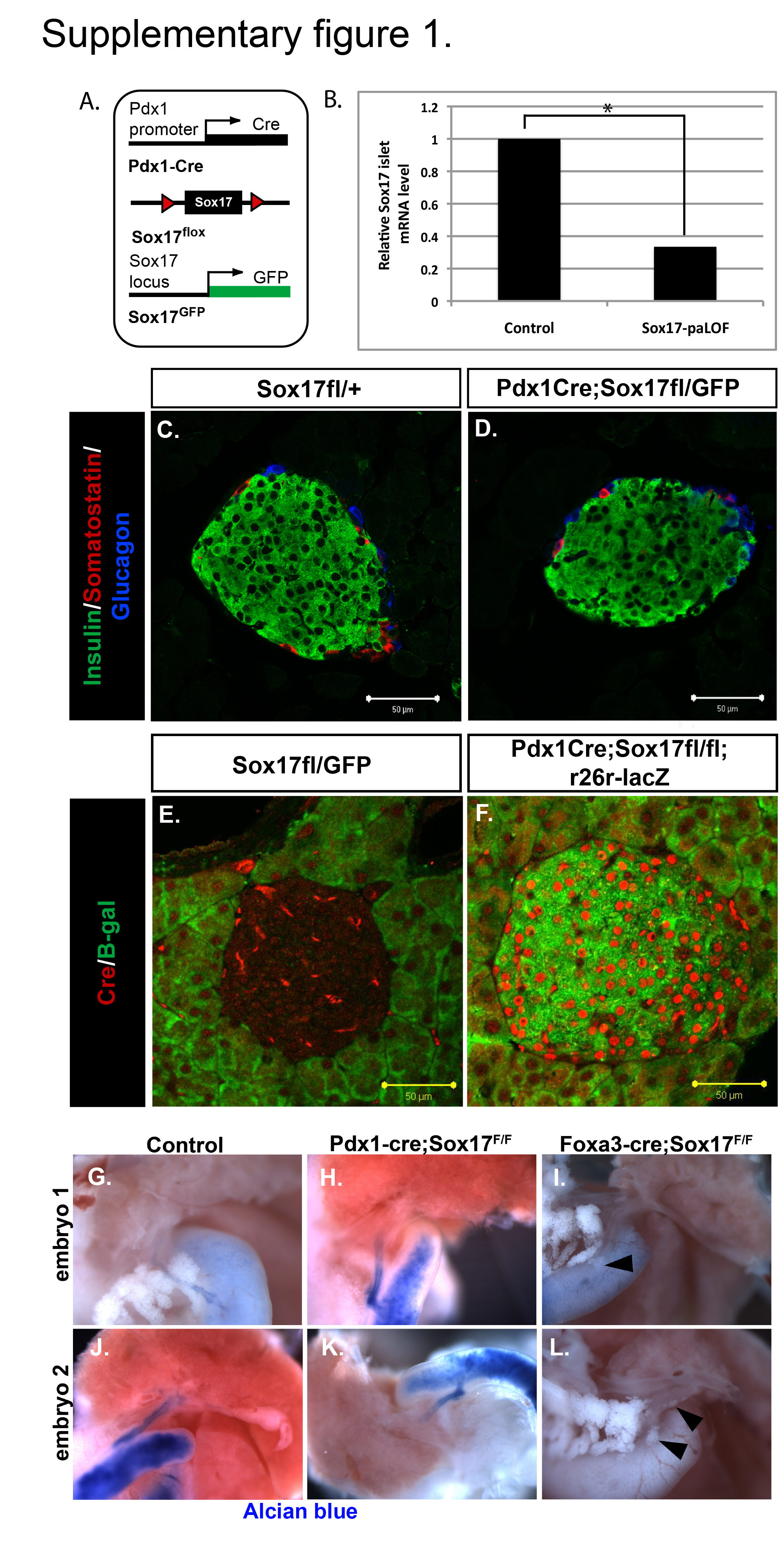

Supplement: Figure S1 — Sox17 is not required for β cell development. A) Schematic representation of the Sox17-paLOF mice. Pdx1-Cre, Sox17fl, and Sox17GFP lines have been previously published [1]–[3]. B) Quantification of fold difference in Sox17 transcript showed significant reduction of Sox17 mRNA levels in Sox17-paLOF islets (asterisk (*) shows p-value≤0.05). Real Time RT-PCR samples were normalized to GAPDH mRNA. (Control mice: Pdx1-Cre;Sox17fl/+, n = 4; Sox17-paLOF mice: Pdx1Cre;Sox17GFP/fl, n = 3) C–D) Immunofluorescence using anti-insulin, -somatostatin, -and glucagon in control and Sox17-paLOF mice show no difference in islet architecture between control and Sox17-paLOF mice. Scale bar: 50 µm. E–F) Immunofluorescence using anti-cre and anti-beta-galactosidase in control and Sox17-paLOF mice containing a r26r-lacZ reporter allele demonstrating Cre expression and efficient recombination in islets. There is significant background staining with the beta galactosidase antibody in the exocrine compartment of the pancreas. Scale bar: 50 µm. G–L) Whole mount images of Pdx1Cre;Sox17fl/fl, Foxa3Cre;Sox17fl/fl and control mice at e16.5. The duodenum of Pdx1Cre;Sox17fl/fl and control animals was injected with alcian blue to provide contrast in the common bile duct. No ectopic pancreas was observed in Pdx1Cre;Sox17fl/fl and control animals, in contrast to Foxa3Cre;Sox17fl/fl mice that have ectopic pancreatic tissue (arrowheads) in the common duct as previously reported [2]. (JPG) [file pone.0104675.s001.jpg]

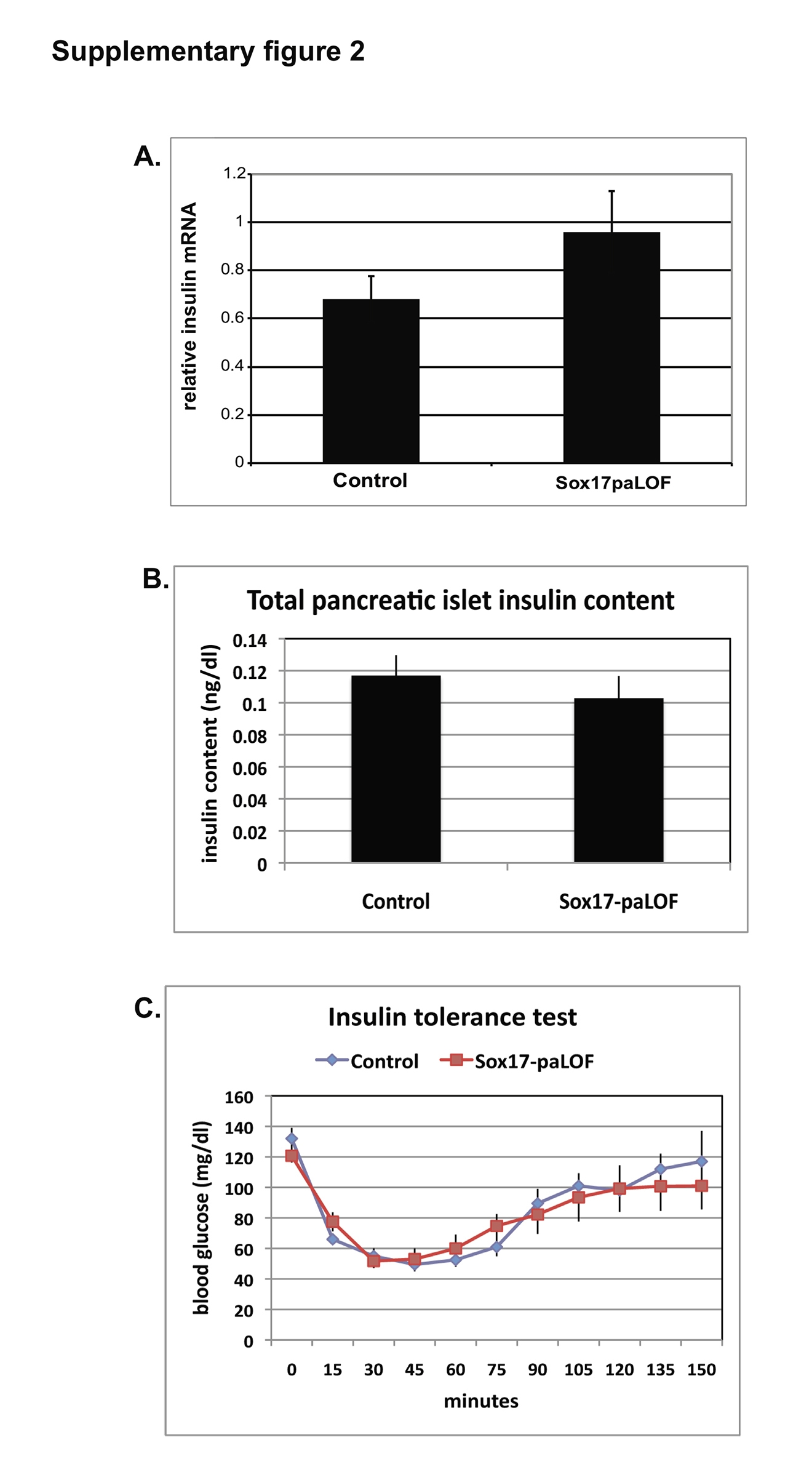

Supplement: Figure S2 — Islet insulin levels and peripheral insulin sensitivity are unaffected in Sox17-paLOF mice. A, B) Isolated islets were isolated from control and Sox17-paLOF mice and were analyzed for total insulin mRNA (Control mice: Sox17fl/+, n = 2, and Pdx1-Cre;Sox17fl/+, n = 1; Sox17-paLOF mice: Pdx1Cre;Sox17GFP/fl, n = 5) and protein (Control mice: Pdx1Cre;Sox17fl/+, n = 4; Sox17-paLOF mice: Pdx1Cre;Sox17GFP/fl, n = 3). C) Animals were tested for peripheral insulin sensitivity by injection of insulin as previously described [4]. There were no changes in insulin sensitivity in Sox17-paLOF mice (Control mice: Sox17+/fl, n = 2; Sox17-paLOF mice: Pdx1Cre;Sox17GFP/fl, n = 4). (JPG) [file pone.0104675.s002.jpg]

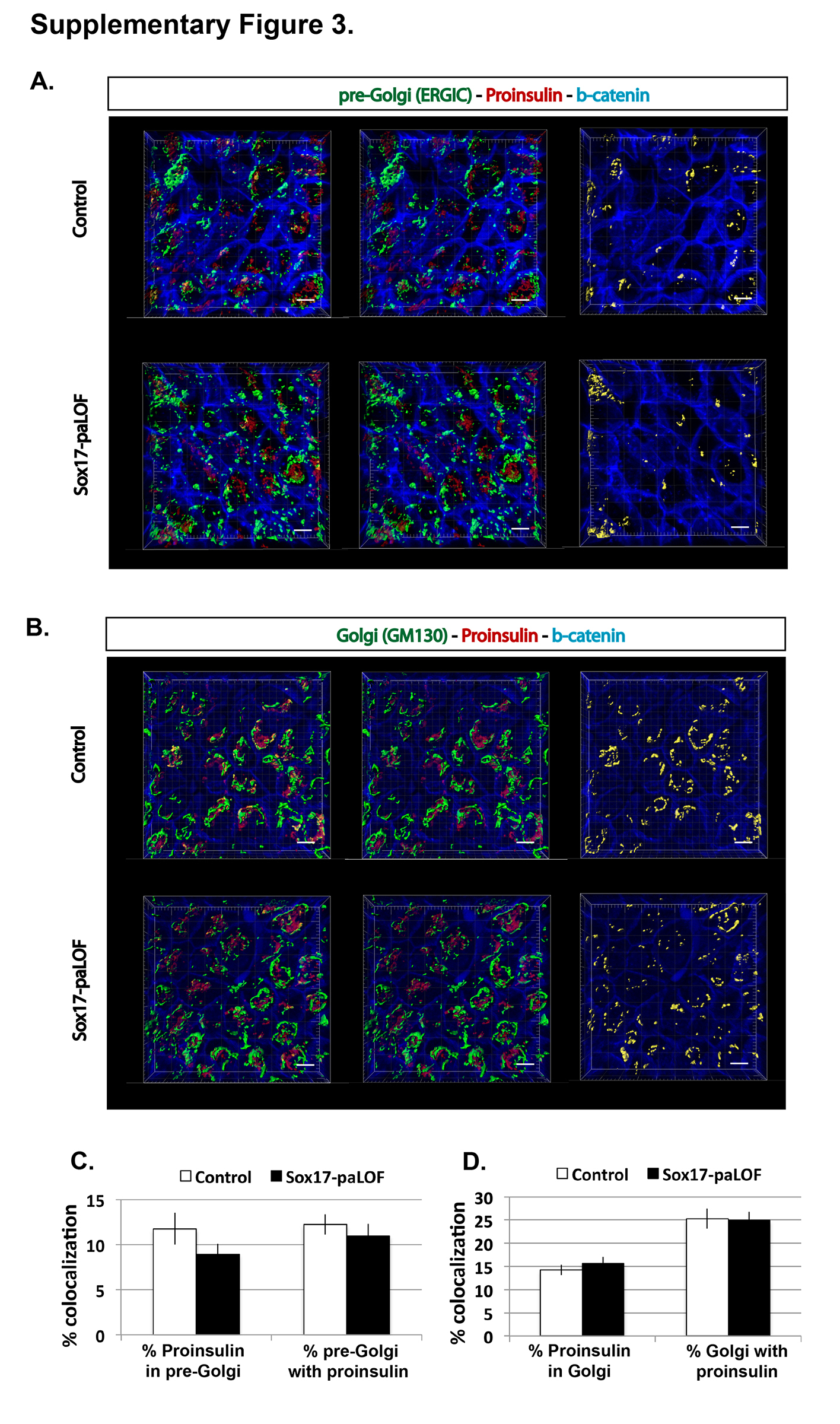

Supplement: Figure S3 — Percent colocalization between proinsulin and organelle markers, and their total regional areas. A, B) Immunofluorescence analysis of proinsulin localization in the pre-Golgi (ERGIC) and Golgi (GM130). Scale bar: 5 µm. C, D) Quantification of A and B indicate that there were no differences found in the levels of colocalization between pre-Golgi and proinsulin, and between Golgi and proinsulin. Quantitation of proinsulin colocalization was performed using Bitplane Imaris software. Control: Sox17fl/+ and Sox17GFP/fl, n = 7 mice, Sox17-paLOF: Pdx1Cre;Sox17GFP/fl, n = 7. 6–10 islets were analyzed per mouse. (JPG) [file pone.0104675.s003.jpg]

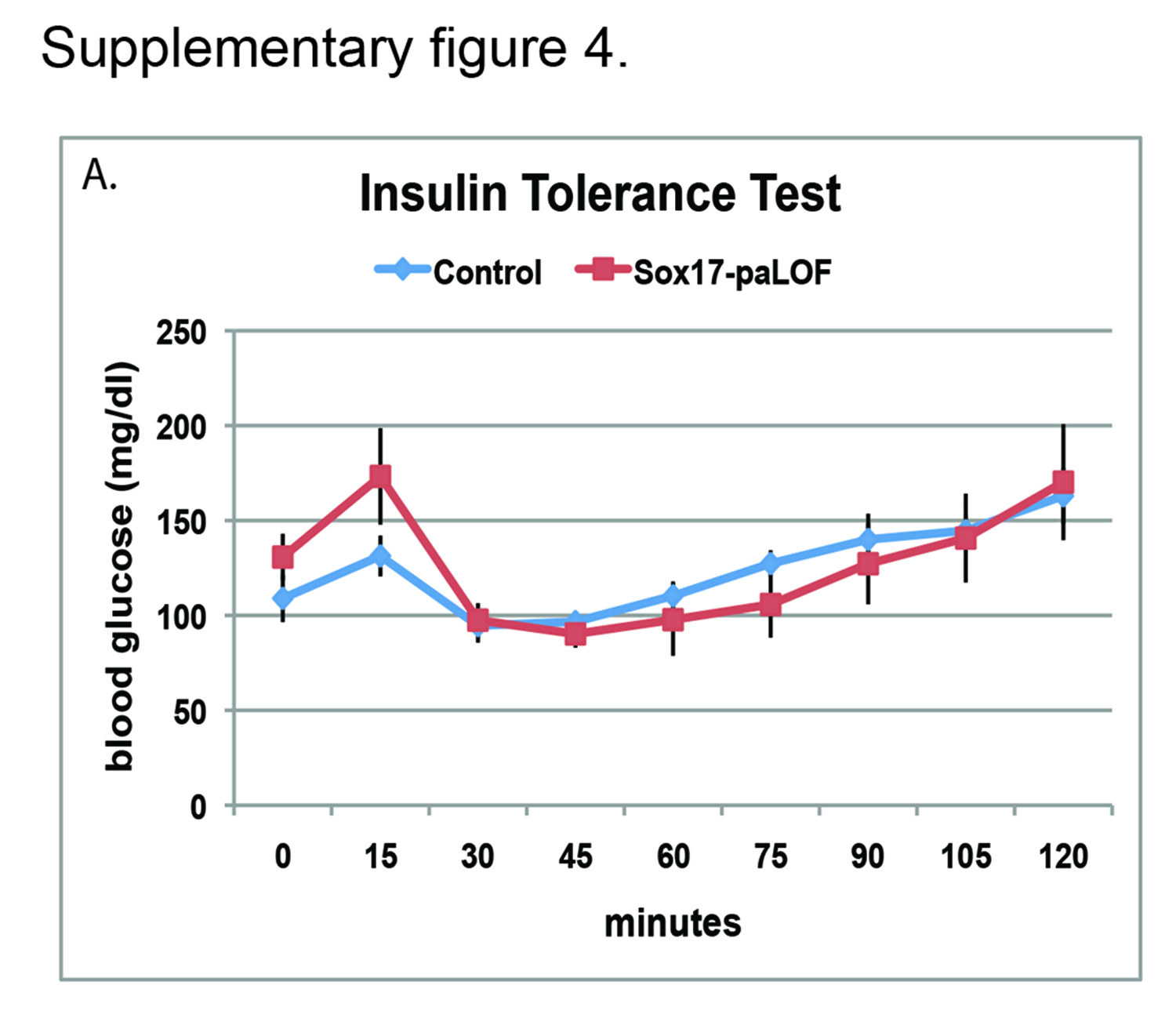

Supplement: Figure S4 — Insulin tolerance test of obese control and Sox17-paLOF mice. Obese animals (26 weeks after high fat diet administration) were fasted for 8–12 hours and intraperitoneally injected with recombinant human insulin (1 U/kg). Control mice: Pdx1Cre;Sox17fl/+, n = 3; Sox17-paLOF mice: Pdx1Cre;Sox17fl/fl, n = 4. Blood glucose levels were measured at the indicated time points. (JPG) [file pone.0104675.s004.jpg]

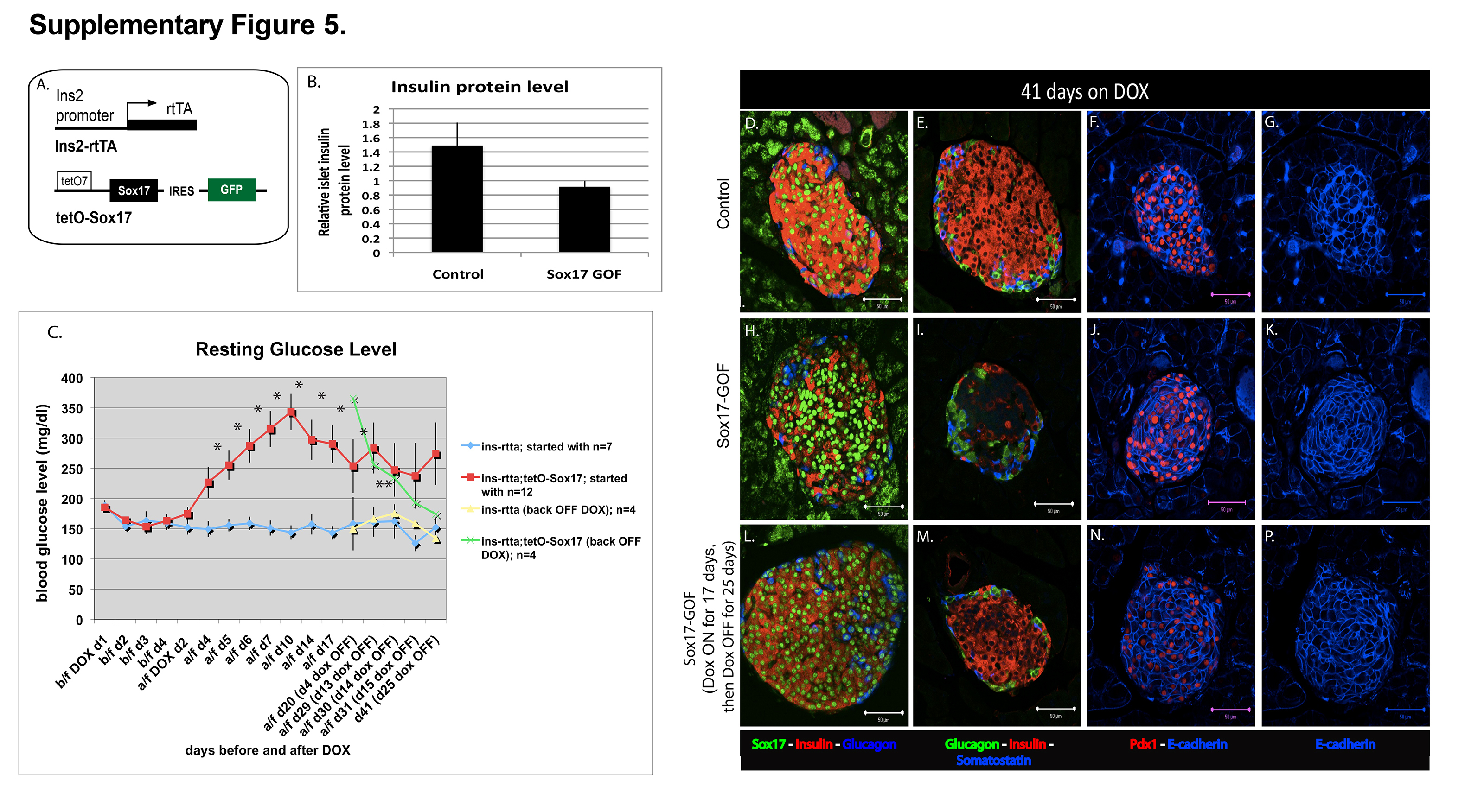

Supplement: Figure S5 — A tetracycline-regulated model for Sox17 overexpression. A) Schematic representation of the Sox17-GOF mice. Ins-rtTA and TetO-Sox17 animals have been described previously [2], [5]–[7]. B) Insulin protein levels are not significantly changed after 24 hours of Sox17 overexpression. C) Hyperglycemia is induced by prolonged dox-inducible Sox17 overexpression, but reverts to normal within 25 days following doxycycline removal (Sox17 off). D–P) Analysis of Sox17, Insulin, Glucagon, Pdx1 and E-cadherin in control, Sox17 overexpressing (Doxycycline ON), and following removal of doxycycline for 25 days. Scale bar: 50 µm. (JPG) [file pone.0104675.s005.jpg]

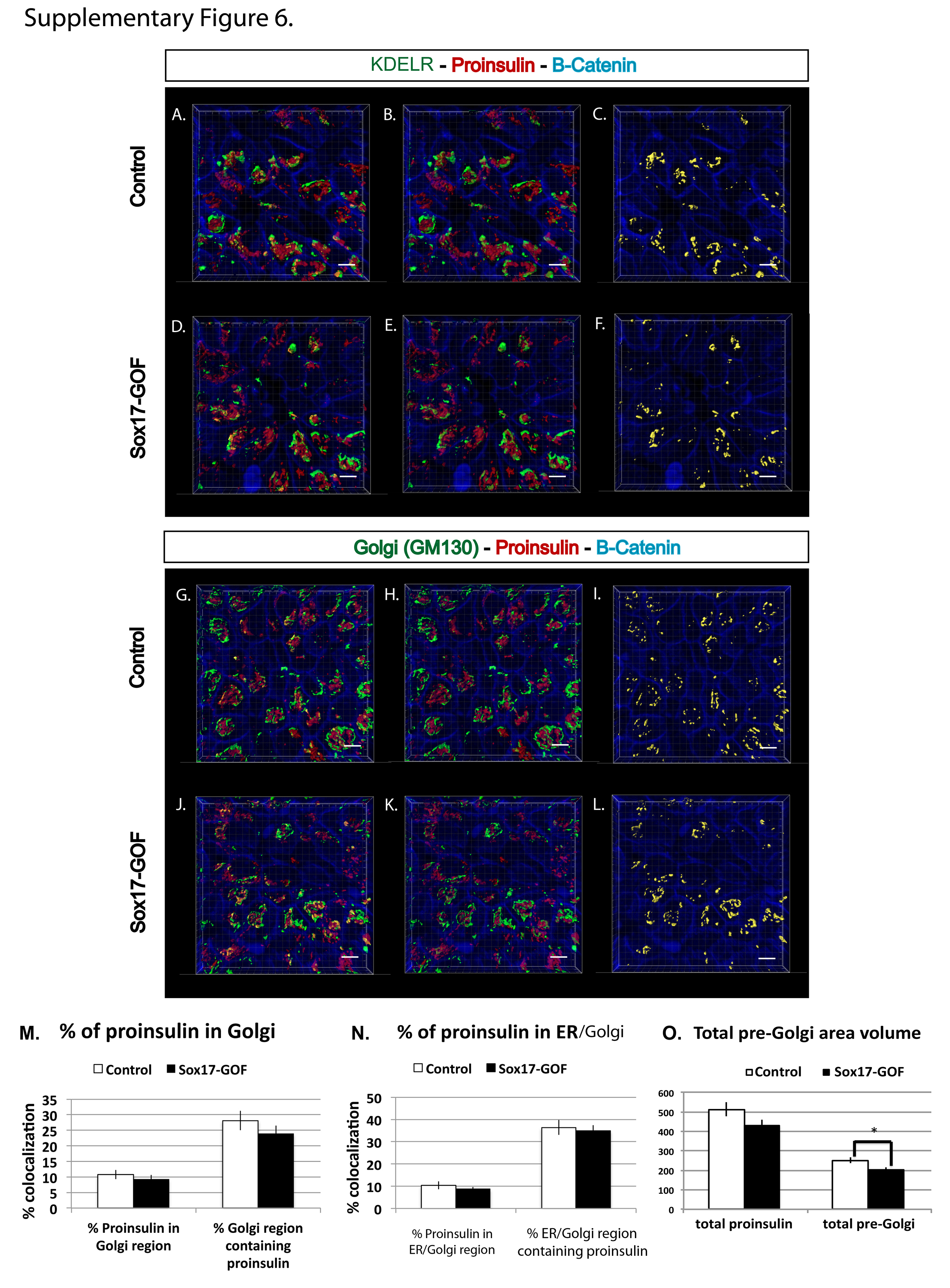

Supplement: Figure S6 — Distribution of proinsulin in the Golgi and ER of Sox17-GOF mice. A–L) Immunofluorescence analysis of proinsulin localization in the ER and Golgi (KDELR) and Golgi only (GM130) in control and Sox17-GOF mice. Scale bar: 5 µm. M and N) Quantification of proinsulin, KDELR, and GM130 staining found no change in the percent of proinsulin in the ER and Golgi O) Quantitation of total proinsulin and pre-Golgi area. (JPG) [file pone.0104675.s006.jpg]

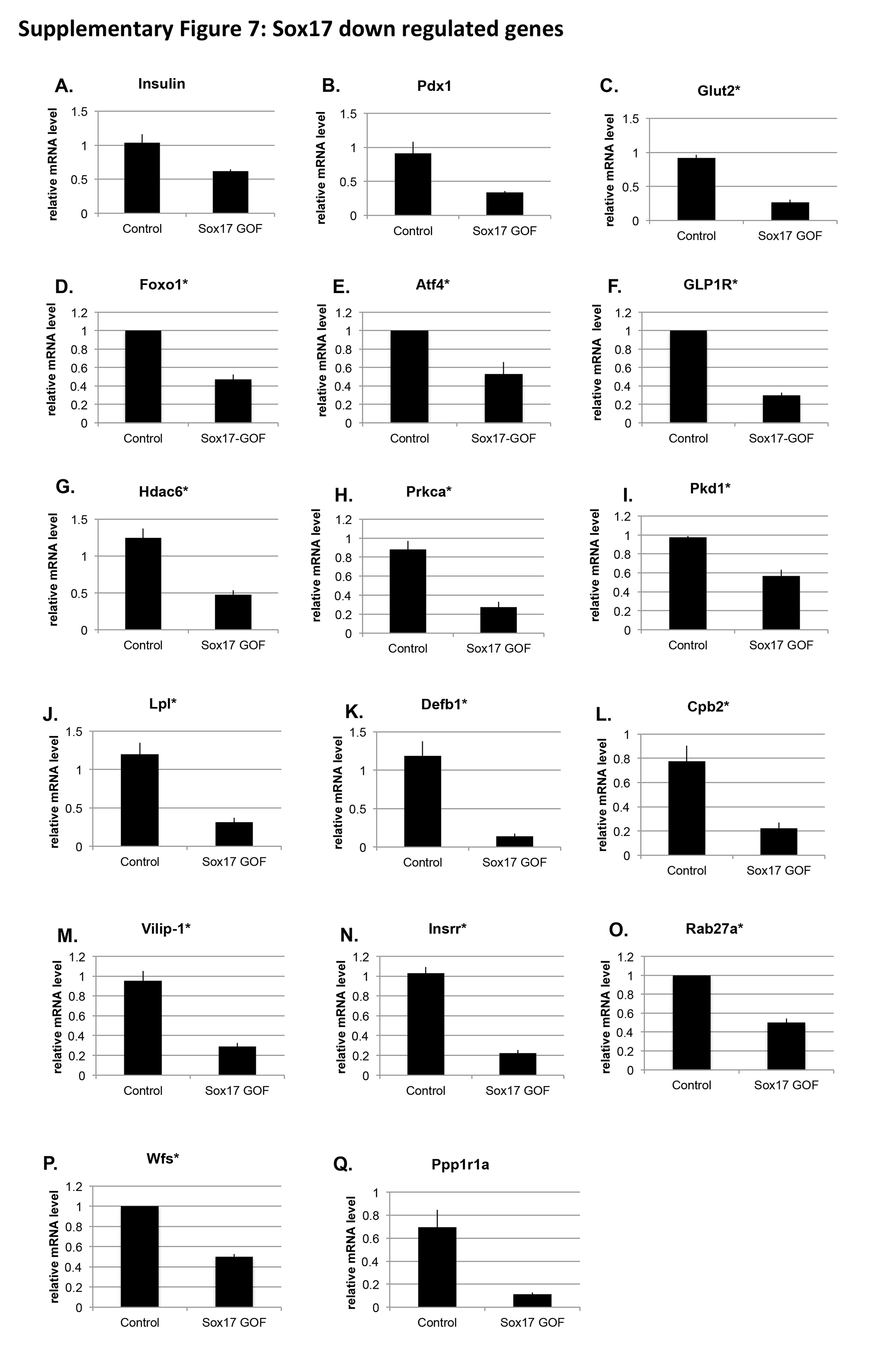

Supplement: Figure S7 — Quantitative RT-PCR validation of down-regulated genes in response to 24 hours of Sox17 overexpression in β cells. A, B) Insulin and Pdx1 mRNA were decreased, but this was not statistically significant. C–P) Glut2, Foxo1, Atf4, GLP1R, Hdac6, Prkca, Pkd1, Lpl, Defb1, Cpb2, Vilip-1, Insrr, Rab27a, Wfs were all significantly down regulated in response to 24 hours of Sox17 overexpression in β bells. Asterisk indicates p-value≤0.05. Q) Ppp1r1a was highly reduced in response to Sox17 overexpression, but this was not statistically significant. (JPG) [file pone.0104675.s007.jpg]

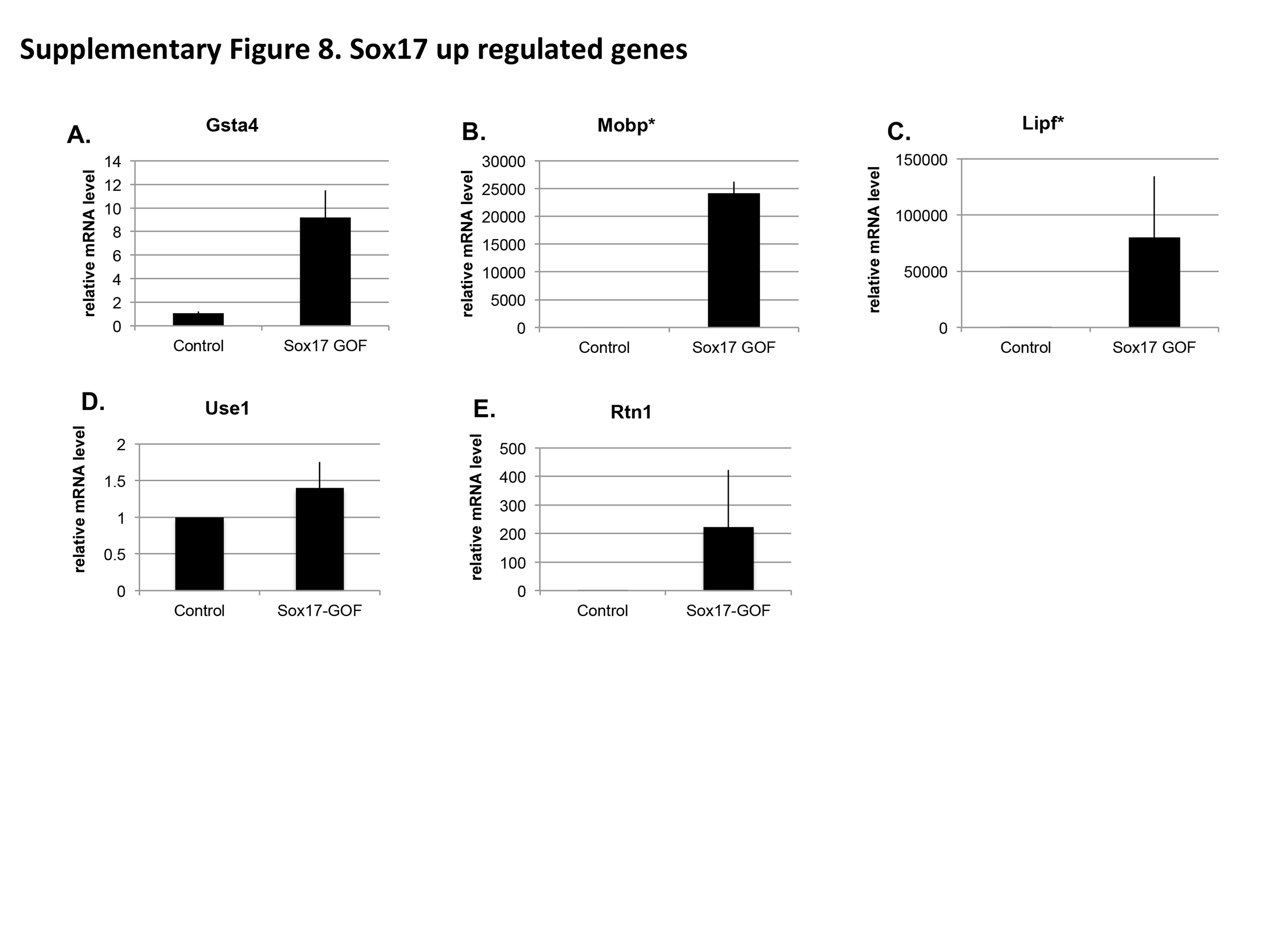

Supplement: Figure S8 — Quantitative RT-PCR validation of up-regulated genes in response to 24 hours of Sox17 overexpression in β cells. A) Gsta4, B) Mobp, C) Lipf, D) Use1, and E) Rrn1 are examples of transcripts that were elevated in β cells in response to 24 hours of Sox17 overexpression. Asterisk indicates p-value≤0.05. (JPG) [file pone.0104675.s008.jpg]

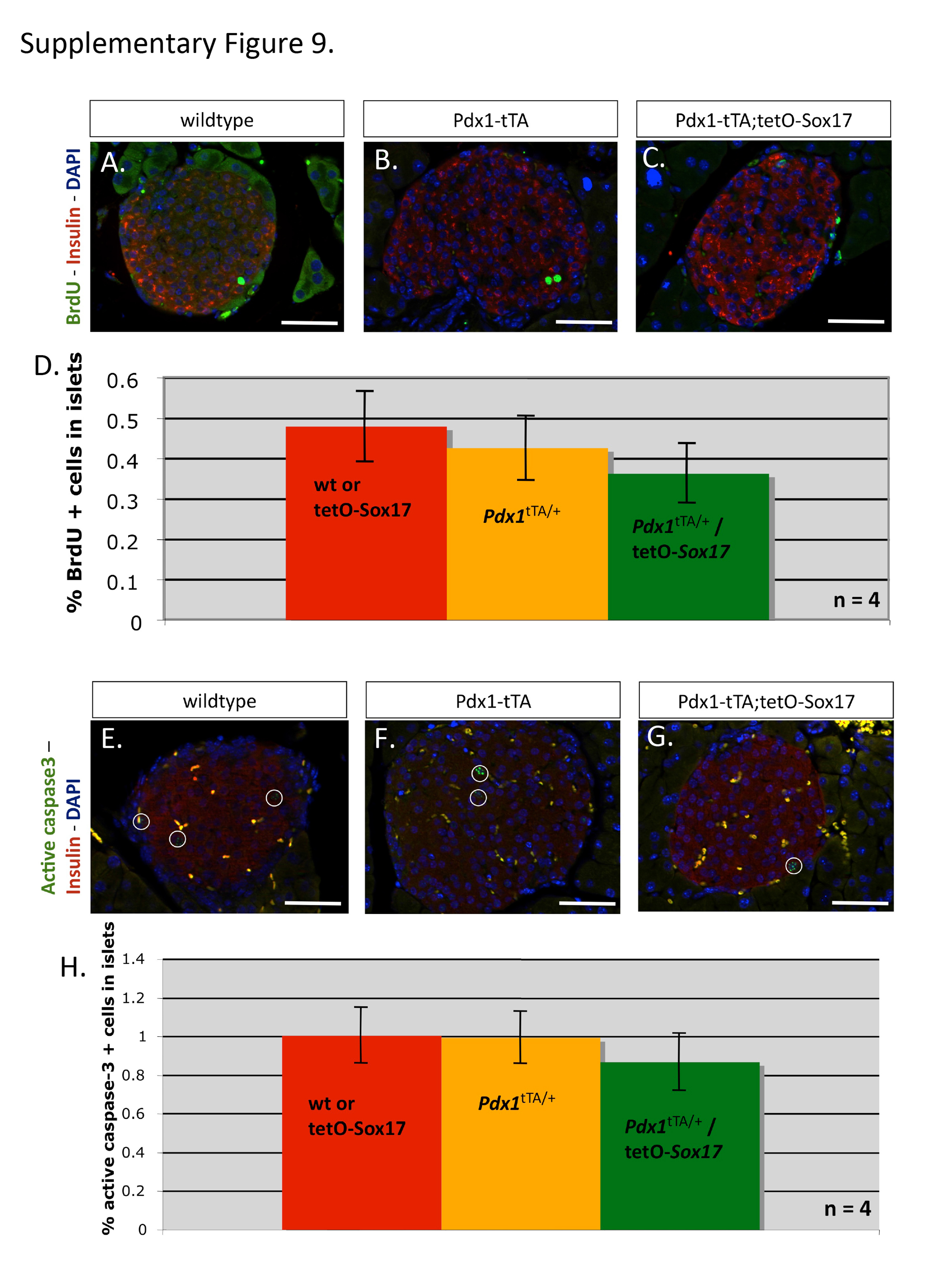

Supplement: Figure S9 — Sox17 expression in MODY4 (Pdx1+/tTA) mice did not alter β cell proliferation or β cell death. A–D) MODY4 (Pdx1+/tTA) mice had comparable levels of BrdU+ cells to both control (Wildtype or tetO-Sox17) and MODY4 (Pdx1+/tTA) mice expressing Sox17. N = 4 animals per genotype. E–H) MODY4 (Pdx1+/tTA) mice had comparable levels of activated caspase3+ cells to both control (Wildtype or tetO-Sox17) and MODY4 (Pdx1+/tTA) mice expressing Sox17. N = 4 animals per genotype. Scale bar: 50 µm. (JPG) [file pone.0104675.s009.jpg]

Supplementary Figure 10.

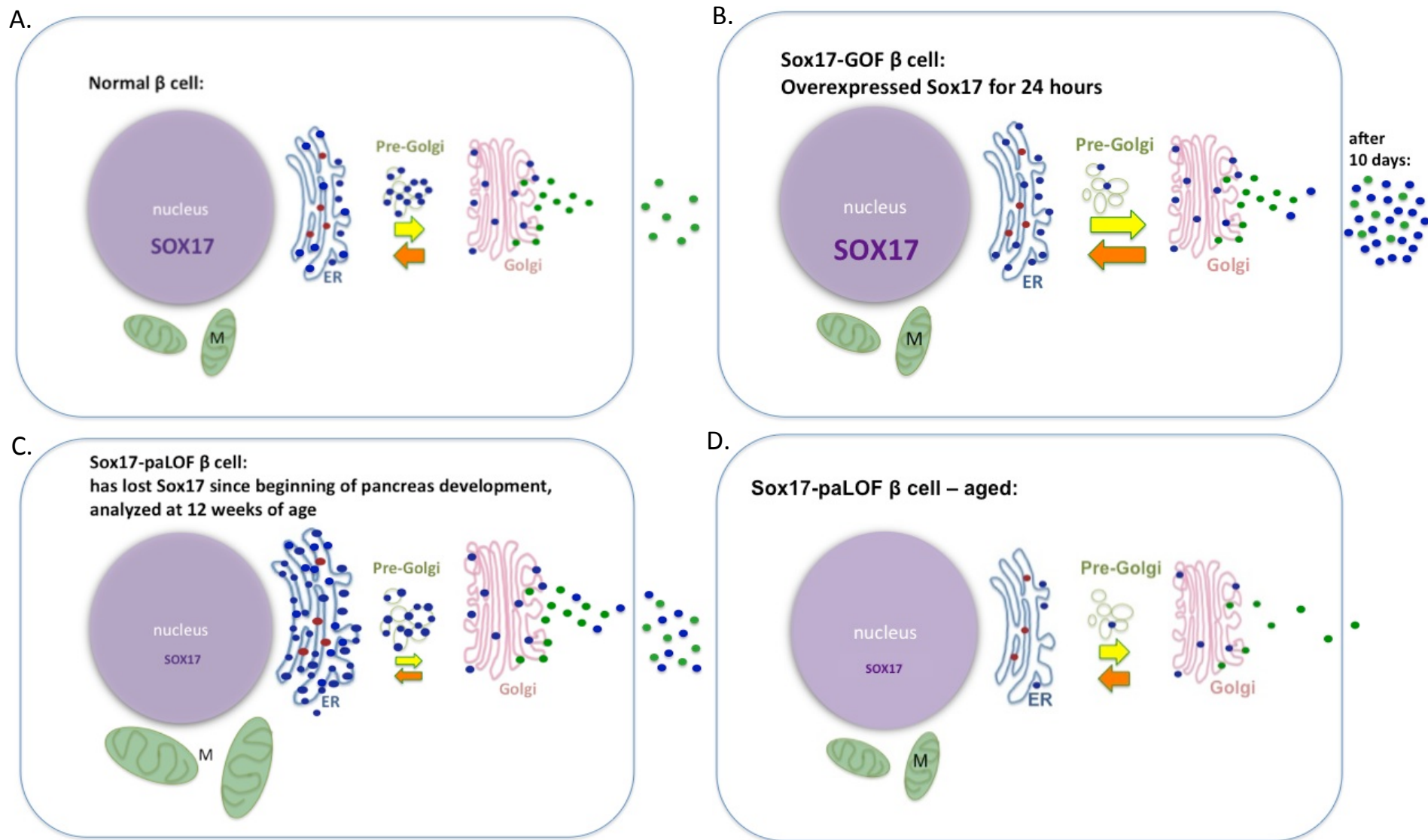

Supplement: Figure S10 — Schematic of β cells in different contexts. A) Normal β cell schematic with normal mitochondria, ER, Pre-Golgi, and Golgi structures and secretory vesicles. Preproinsulin containing secretory vesicle is in red, proinsulin containing secretory vesicle is in blue, and insulin + C-peptide containing secretory vesicle is in green. Arrows show the anterograde (yellow arrow) and retrograde (orange arrow) movements of the vesicles. B) Sox17 overexpressing β cell schematic. The cell had less amount of proinsulin vesicles trafficking through pre-Golgi region. Over time, a 4-fold increased in unprocessed proinsulin is found in the plasma. C) Prediabetic Sox17-paLOF β cell schematic at 12 weeks old. The cell had dilated and distended secretory organelles, accumulated proinsulin, and a trend of increased secretion of proinsulin. D) Diabetic Sox17-paLOF β cell schematic at 1.5 years old. Some of the ER were dispersed and dilated and the cells had less insulin in the secretory organelles and granules. Overall, the mice had reduced β cell mass. (PDF) [file pone.0104675.s010.pdf]
