# Supplementary material for: Sox17 Regulates Insulin Secretion in the Normal and Pathologic Mouse β Cell
Source: PLoS One. 2014 Aug 21;9(8):e104675. doi: 10.1371/journal.pone.0104675 (PMC4140688; doi:10.1371/journal.pone.0104675)
Supplement: File S1 — Supporting tables and references. Table S1, Primary and Secondary Antibodies. Table S2, Transcripts that were changed in Sox17-GOF islets. List of transcripts change by >1.55 fold in Sox17-GOF islets. Red highlighted genes were upregulated, blue highlighted genes were downregulated. Table S3, Gene ontology analysis of biological pathways and processes associated with SOX17 regulated transcripts. List of gene ontology of the biological process that are involved in the Sox17-misregulated genes cluster in the Sox17-GOF islets microarray by 1.30 fold change and above (upregulated genes cluster in red, downregulated genes cluster in blue). Table S4, PCR primers for microarray genes validation. (DOCX) [file pone.0104675.s011.docx]

**Table S1:** Primary and Secondary Antibodies

| **Primary Antibody** | **Source** | **Dilution** |
| --- | --- | --- |
| Rat anti-BrdU | Abcam | 1:100 |
| Guinea Pig anti-Sox17 | Whitsett Lab | 1:1000 |
| Mouse anti-FoxA2 | Novus Biologicals | 1:500 |
| Mouse anti-proinsulin | R&D | 1:500 |
| Rabbit anti-Cre | Novagen | 1:5000 |
| Chicken anti-BGal | Abcam | 1:1000 |
| Rat anti-Insulin | R&D | 1:200 |
| Goat anti-Somatostatin | Santa Cruz | 1:200 |
| Guinea Pig anti-Glucagon | Linco | 1:1000 |
| Rabbit anti-active Caspase 3 | Cell Signaling | 1:100 |
| Mouse anti-KDELR | Enzo | 1:100 |
| Rabbit anti-ERGIC | Sigma | 1:200 |
| Mouse anti-E-Cadherin | BD Biosciences | 1:500 |
| Mouse anti-GM130 | BD Transduction | 1:100 |
| Goat anti-Glut2 | Santa Cruz | 1:1000 |
| Mouse anti-Nkx2.2 | 74.5A5-C | 1:100 |
| Goat anti-Pdx1 | Abcam | 1:100 |
| Goat anti-Hnf1B | Santa Cruz | 1:100 |
| Rabbit anti-B-catenin | Santa Cruz | 1:100 |
| Guinea Pig anti-Insulin | DAKO | 1:1000 |
| **Secondary Antibody** | **Source** | Dilution |
| Goat anti-mouse IgG2a 549 | Jackson Immuno | 1:400 |
| Goat anti-chicken Biotin | Jackson Immuno | 1:200 |
| Goat anti-mouse IgG1 488 | Jackson Immuno | 1:400 |
| Goat anti-rabbit 488 | Invitrogen | 1:400 |
| Goat anti-mouse Biotin | Jackson Immuno | 1:200 |
| Goat anti-guinea pig Cy5 | Jackson Immuno | 1:200 |
| Goat anti-rabbit Cy5 | Jackson Immuno | 1:200 |
| Donkey anti-rabbit Cy3 | Jackson Immuno | 1:200 |
| Donkey anti-rat 546 | Invitrogen | 1:200 |
| Donkey anti-goat 549 | Jackson Immuno | 1:200 |
| Donkey anti-rabbit 488 | Invitrogen | 1:200 |
| Donkey anti-guinea pig Cy5 | Jackson Immuno | 1:200 |
| Donkey anti-guine pig 549 | Jackson Immuno | 1:200 |
| Donkey anti-guine pig biotin | Jackson Immuno | 1:200 |
| Donkey anti-goat 488 | Jackson Immuno | 1:200 |
| Donkey anti-goat biotin | Invitrogen | 1:200 |
| Donkey anti-mouse 647 | Invitrogen | 1:200 |
| Donkey anti-mouse Cy3 | Jackson Immuno | 1:200 |
| **Conjugates/Nuclear Dyes** |  |  |
| Strepavidin cy5 | Jackson Immuno | 1/500 |
| TOPRO3 | Jackson Immuno | 1/20,000 |
| DRAQ5 | Cell Signaling | 1/10,000 |
| TSA KIT, tyr 488 | Invitrogen | 1:200 |
| TSA KIT, tyr 549 | Invitrogen | 1:200 |

**Table S1.** Primary and Secondary Antibodies.

**Table S2: Transcripts that were changed in Sox17-GOF islets.**

| **Genbank** | **Fold Change** | **P-value** | **Common** |
| --- | --- | --- | --- |
| ENSMUST00000025680 | 29.45 | 0.0356 | Lipf |
| XM_917532 | 27.57 | 1.66E-05 | EG640530 |
| NM_008614 | 21.66 | 0.00165 | Mobp |
| ENSMUST00000034903 | 17.76 | 0.00606 | Gsta4 |
| NM_019790 | 13.95 | 0.00112 | Tmeff2 |
| BC055924 | 13.93 | 0.00831 | EG545886 |
| AF498319 | 11.11 | 3.95E-05 | Rgs13 |
| BC023359 | 10.09 | 0.00228 | 3632451O06Rik |
| NM_013927 | 9.731 | 0.00064 | Cngb3 |
| NM_175647 | 7.219 | 0.00273 | Dmrta1 |
| NM_145629 | 6.465 | 0.000132 | Pls3 |
| BC048664 | 6.401 | 0.0203 | 1700016D06Rik |
| ENSMUST00000014248 | 6.234 | 0.0462 | Sval2 |
| M65237 | 6.227 | 0.0246 | Mug-ps1 |
| NM_026713 | 6.055 | 0.0265 | Mogat1 |
| NM_177747 | 5.989 | 0.000235 | Zfp711 |
| NM_133348 | 5.557 | 0.00198 | Acot7 |
| ENSMUST00000099091 | 5.332 | 6.18E-05 | Gm410 |
| NM_009037 | 5.249 | 9.30E-06 | Rcn1 |
| NM_134022 | 5.221 | 0.0024 | 6330403K07Rik |
| NM_172781 | 5.206 | 0.0462 | Klhl4 |
| NM_019754 | 5.117 | 0.00182 | Tagln3 |
| NM_009801 | 4.937 | 0.00438 | Car2 |
| NM_080448 | 4.575 | 0.00224 | Srgap3 |
| NM_008278 | 4.511 | 0.0137 | Hpgd |
| NM_175465 | 4.459 | 0.00123 | Sestd1 |
| NM_008885 | 4.426 | 0.000901 | Pmp22 |
| NM_175771 | 4.37 | 0.00805 | Tmem47 |
| NM_011807 | 4.339 | 0.00296 | Dlg2 |
| NM_177373 | 4.209 | 0.00233 | Ppfia2 |
| NM_010408 | 4.145 | 0.00216 | Hcn1 |
| ENSMUST00000085469 | 4.066 | 0.00618 | Pik3cg |
| NM_153457 | 4.037 | 0.0176 | Rtn1 |
| NM_008594 | 4.014 | 0.00237 | Mfge8 |
| NM_138595 | 3.958 | 0.0192 | Gldc |
| NM_011348 | 3.824 | 0.000452 | Sema3e |
| ENSMUST00000064054 | 3.821 | 0.0149 | Syt1 |
| XM_146632 | 3.723 | 0.00103 | 9030420J04Rik |
| NM_027997 | 3.721 | 0.000584 | Serpina9 |
| NM_011254 | 3.718 | 0.000254 | Rbp1 |
| NM_013930 | 3.683 | 0.00191 | Aass |
| NM_019703 | 3.605 | 0.00272 | Pfkp |
| NM_019656 | 3.586 | 0.00143 | Tspan6 |
| NM_019641 | 3.54 | 2.97E-05 | Stmn1 |
| NM_010251 | 3.515 | 0.0172 | Gabra4 |
| NM_027168 | 3.498 | 0.00134 | Hddc2 |
| ENSMUST00000030303 | 3.493 | 0.00774 | Cyp2j6 |
| NM_019641 | 3.488 | 9.45E-05 | Stmn1 |
| NM_008481 | 3.432 | 0.000217 | Lama2 |
| NM_001011875 | 3.419 | 0.024 | Rnase12 |
| NM_173379 | 3.4 | 0.000368 | Leprel1 |
| NM_013657 | 3.395 | 0.00456 | Sema3c |
| NM_009807 | 3.341 | 0.00549 | Casp1 |
| NM_025760 | 3.303 | 0.0254 | Ptplad2 |
| NM_001082976 | 3.288 | 0.00142 | Tc2n |
| NM_080448 | 3.273 | 0.0118 | Srgap3 |
| NM_029942 | 3.273 | 0.00056 | Prelid2 |
| NM_029447 | 3.19 | 0.00042 | Nln |
| NM_181414 | 3.187 | 0.0058 | Pik3c3 |
| ENSMUST00000040423 | 3.152 | 0.0204 | Cd59a |
| AK046516 | 3.132 | 0.00687 | ENSMUSG00000071543 |
| ENSMUST00000107802 | 3.11 | 0.00124 | Trim59 |
| NM_080462 | 3.108 | 0.00883 | Hnmt |
| AF184981 | 3.073 | 0.0402 | Fmo2 |
| ENSMUST00000029876 | 3.068 | 0.0018 | Calb1 |
| NM_198702 | 3.058 | 0.00054 | Lphn3 |
| NM_010807 | 3.034 | 0.0218 | Marcksl1 |
| NM_010634 | 3.033 | 0.00105 | Fabp5 |
| NM_018863 | 3.002 | 0.0238 | Pdyn |
| BC051224 | 3 | 0.0172 | A530053G22Rik |
| NM_001042592 | 2.992 | 0.0478 | Arrdc4 |
| ENSMUST00000004497 | 2.948 | 0.00089 | Large |
| NM_010597 | 2.922 | 0.00749 | Kcnab1 |
| NM_146100 | 2.918 | 0.000279 | Ina |
| ENSMUST00000046513 | 2.902 | 0.00286 | Phyhipl |
| NM_007697 | 2.889 | 0.0137 | Chl1 |
| BC002008 | 2.871 | 2.00E-05 | Fabp5 |
| NM_001033773 | 2.856 | 0.0351 | Ube2u |
| NM_001081128 | 2.833 | 0.00035 | Mtr |
| NM_001033331 | 2.807 | 0.0296 | Gas2l3 |
| NM_178779 | 2.803 | 0.00601 | Rnf152 |
| NM_001009935 | 2.786 | 0.0377 | Txnip |
| BC055351 | 2.782 | 0.00489 | Hn1l |
| NM_144855 | 2.759 | 0.0175 | Cbs |
| ENSMUST00000039366 | 2.745 | 0.00467 | Kcnh8 |
| NM_019691 | 2.745 | 0.000159 | Gria4 |
| NM_024279 | 2.738 | 0.0112 | 1700094C09Rik |
| NM_029619 | 2.724 | 0.00179 | Msrb2 |
| ENSMUST00000033554 | 2.72 | 0.0178 | Gpr165 |
| NM_026167 | 2.706 | 0.0107 | Klhl13 |
| ENSMUST00000007340 | 2.704 | 0.0014 | Atp12a |
| NM_207685 | 2.701 | 0.00626 | Elavl2 |
| NM_013709 | 2.674 | 0.000149 | Sh3yl1 |
| ENSMUST00000081721 | 2.659 | 0.000244 | Ezh2 |
| NM_011035 | 2.658 | 0.013 | Pak1 |
| NM_172805 | 2.655 | 0.00654 | Kcnh5 |
| NM_018779 | 2.647 | 0.0405 | Pde3a |
| NM_027971 | 2.619 | 0.0132 | Serpinb12 |
| NM_001033346 | 2.612 | 0.0106 | Lrrc55 |
| NM_172824 | 2.598 | 0.0016 | Ccdc14 |
| NM_029466 | 2.574 | 0.00589 | Arl5b |
| ENSMUST00000019290 | 2.561 | 0.00513 | Cacng2 |
| BC100412 | 2.553 | 0.00586 | 1700001E04Rik |
| NM_019631 | 2.526 | 0.0471 | Tmem45a |
| NM_172671 | 2.526 | 0.0002 | Lgr4 |
| NM_001001804 | 2.519 | 0.00706 | Abhd7 |
| NM_001029930 | 2.517 | 0.00418 | ENSMUSG00000068790 |
| BC117712 | 2.514 | 0.000213 | Clec12b |
| NM_021099 | 2.482 | 0.00177 | Kit |
| ENSMUST00000078021 | 2.468 | 0.0145 | Glmn |
| NM_172310 | 2.461 | 0.0409 | Tarsl2 |
| NM_178005 | 2.442 | 0.00535 | Lrrtm2 |
| NM_008604 | 2.442 | 0.00172 | Mme |
| BC049669 | 2.428 | 5.64E-05 | 1700047I17Rik1 |
| BC049669 | 2.428 | 5.64E-05 | 1700047I17Rik1 |
| NM_201371 | 2.423 | 0.0186 | Prmt8 |
| BC037216 | 2.397 | 0.00309 | Lrrtm4 |
| NM_153124 | 2.393 | 0.0103 | St8sia5 |
| NM_001083897 | 2.391 | 0.00386 | Mpzl1 |
| NM_023245 | 2.375 | 0.0079 | Palmd |
| NM_198302 | 2.374 | 0.0123 | Rbm11 |
| NM_028719 | 2.37 | 0.00957 | Cpne4 |
| BC050850 | 2.359 | 0.00977 | AK129302 |
| NM_007675 | 2.357 | 0.0206 | Ceacam10 |
| NM_028651 | 2.357 | 0.0115 | Tmtc4 |
| NM_153587 | 2.355 | 0.0248 | Rps6ka5 |
| ENSMUST00000070522 | 2.355 | 0.00154 | Plod2 |
| NM_011943 | 2.354 | 0.00135 | Map2k6 |
| NM_153163 | 2.35 | 0.0056 | Cadps2 |
| NM_013540 | 2.34 | 0.00106 | Gria2 |
| NM_026139 | 2.338 | 0.0061 | Armcx2 |
| XM_001479202 | 2.328 | 0.0112 | LOC100047943 |
| NM_198422 | 2.324 | 0.00589 | Paqr3 |
| NM_029001 | 2.312 | 0.00623 | Elovl7 |
| BC060180 | 2.309 | 0.00646 | Ccng2 |
| NM_133239 | 2.309 | 0.000543 | Crb1 |
| EF651808 | 2.291 | 0.00278 | Hn1l |
| NM_009121 | 2.288 | 0.000817 | Sat1 |
| NM_178673 | 2.284 | 0.014 | Fstl5 |
| NM_177173 | 2.277 | 0.0142 | A830018L16Rik |
| NM_028979 | 2.273 | 0.000678 | Cyp2j9 |
| NM_010315 | 2.271 | 0.00905 | Gng2 |
| NM_146140 | 2.265 | 0.00681 | Tram1l1 |
| NM_001081121 | 2.243 | 0.027 | 4931429I11Rik |
| EF651808 | 2.241 | 0.00108 | Hn1l |
| NM_001083628 | 2.238 | 0.000428 | AK220484 |
| NM_010164 | 2.234 | 0.00572 | Eya1 |
| NM_007913 | 2.228 | 0.0139 | Egr1 |
| ENSMUST00000075853 | 2.217 | 0.0298 | Cks2 |
| NM_001031664 | 2.209 | 0.0172 | Nudt10 |
| AK129372 | 2.209 | 0.00784 | 9430031J16Rik |
| NM_172256 | 2.208 | 0.00251 | Dync2li1 |
| ENSMUST00000035038 | 2.204 | 0.00423 | Faim |
| BC016222 | 2.201 | 0.00273 | LOC544988 |
| NM_001099298 | 2.2 | 0.0211 | Scn2a1 |
| NM_145575 | 2.198 | 0.00136 | Cald1 |
| NM_028876 | 2.192 | 0.0163 | Tmed5 |
| NM_134072 | 2.189 | 0.00578 | Akr1c14 |
| NM_028810 | 2.184 | 0.0282 | Rnd3 |
| NM_008633 | 2.17 | 0.00062 | Mtap4 |
| NM_010237 | 2.162 | 0.0357 | Frk |
| NM_013754 | 2.162 | 0.0192 | Insl6 |
| NM_028665 | 2.158 | 0.00374 | Ankrd42 |
| NM_177290 | 2.15 | 0.0394 | Itgb8 |
| ENSMUST00000030684 | 2.14 | 0.00432 | Gnl2 |
| NM_026470 | 2.135 | 0.00448 | Spata6 |
| NM_001037725 | 2.135 | 4.07E-05 | Als2cr13 |
| ENSMUST00000002926 | 2.134 | 0.00151 | Pla1a |
| NM_133227 | 2.133 | 0.0154 | Nup155 |
| ENSMUST00000028369 | 2.127 | 0.0305 | Dapl1 |
| ENSMUST00000053950 | 2.125 | 0.0244 | Lrrc28 |
| EF651808 | 2.12 | 0.00233 | Hn1l |
| NM_017377 | 2.119 | 0.00507 | B4galt2 |
| ENSMUST00000099343 | 2.118 | 0.0403 | Nr2c1 |
| DQ112091 | 2.117 | 0.00164 | B230118H07Rik |
| NM_023249 | 2.103 | 0.0172 | Ypel1 |
| BC100412 // BC100412 | 2.102 | 0.000781 | 1700001E04Rik // 1700001E04Rik |
| ENSMUST00000057866 | 2.101 | 0.0291 | Nrsn1 |
| ENSMUST00000029423 | 2.098 | 0.0155 | Serpini1 |
| NM_032465 | 2.094 | 0.00344 | Cd96 |
| AB073967 | 2.09 | 0.00404 | 1100001E04Rik |
| NM_001024706 | 2.08 | 0.00767 | EG432825 |
| BC129903 | 2.072 | 0.0127 | Fgd4 |
| EF651808 | 2.053 | 0.00224 | Hn1l |
| NM_008083 | 2.048 | 0.0377 | Gap43 |
| NM_009923 | 2.048 | 0.0257 | Cnp |
| NM_009199 | 2.042 | 0.00277 | Slc1a1 |
| NM_026825 | 2.037 | 0.012 | Lrrc16 |
| BC035305 | 2.034 | 0.00588 | Aig1 |
| NM_181579 | 2.028 | 0.0136 | Pof1b |
| ENSMUST00000114292 | 2.027 | 0.0474 | Cadm2 |
| BC032970 | 2.026 | 0.00122 | 2810026P18Rik |
| NM_001043335 | 2.022 | 0.0199 | Eml1 |
| NM_010882 | 2.016 | 0.000483 | Ndn |
| NM_177591 | 2.014 | 0.00468 | Igsf1 |
| NM_001098230 | 2.005 | 0.0129 | Ppm2c |
| NM_019958 | 2.002 | 0.00457 | Rgs17 |
| NM_001081664 | 1.998 | 0.0102 | 4833423E24Rik |
| ENSMUST00000052431 | 1.996 | 0.0161 | Armcx6 |
| NM_027409 | 1.994 | 0.0266 | Mospd1 |
| ENSMUST00000075853 | 1.992 | 0.0287 | Cks2 |
| NM_145928 | 1.99 | 0.0211 | Tspan14 |
| NM_008576 | 1.984 | 0.0433 | Abcc1 |
| XR_032130 | 1.981 | 0.0146 | LOC667519 |
| NM_145743 | 1.978 | 0.0181 | Lace1 |
| NM_023598 | 1.97 | 0.0278 | Arid5b |
| NM_008485 | 1.97 | 0.0101 | Lamc2 |
| ENSMUST00000034801 | 1.964 | 0.0364 | Bckdhb |
| NM_175393 | 1.964 | 0.0122 | 4930555G01Rik |
| NM_175393 | 1.964 | 0.0122 | 4930555G01Rik |
| NM_027934 | 1.961 | 0.000703 | Rnf180 |
| NM_001083628 | 1.96 | 0.00932 | AK220484 |
| NM_001081257 | 1.96 | 0.00124 | LOC545291 |
| NM_009255 | 1.948 | 0.0428 | Serpine2 |
| NM_008585 | 1.943 | 0.000297 | Mep1a |
| NM_027569 | 1.938 | 0.00124 | Spag9 |
| NM_020574 | 1.936 | 0.0376 | Kcne3 |
| NM_008017 | 1.934 | 0.0141 | Smc2 |
| ENSMUST00000040056 | 1.934 | 0.00436 | Ppfibp2 |
| NM_030165 | 1.931 | 0.00906 | Galnact2 |
| NM_145940 | 1.927 | 0.00287 | Wipi1 |
| ENSMUST00000057019 | 1.917 | 0.00807 | Agtrl1 |
| NM_023732 | 1.917 | 0.00754 | Abcb6 |
| NM_146201 | 1.913 | 0.0233 | Zfp553 |
| NM_153195 | 1.91 | 0.0385 | Fbxo7 |
| NM_177742 | 1.9 | 0.0292 | Triml1 |
| NM_011883 | 1.887 | 0.0291 | Rnf13 |
| NM_013751 | 1.887 | 0.00922 | Hrasls |
| NM_173760 | 1.881 | 0.0251 | Hisppd1 |
| ENSMUST00000049681 | 1.88 | 0.0466 | Itgbl1 |
| NM_054088 | 1.88 | 0.00532 | Pnpla3 |
| NM_025816 | 1.879 | 0.000175 | Tax1bp1 |
| ENSMUST00000008179 | 1.878 | 0.0061 | Mid1ip1 |
| NM_027571 | 1.877 | 0.0477 | P2ry12 |
| NM_026115 | 1.877 | 0.00982 | Hat1 |
| NM_172770 | 1.875 | 0.00825 | Ttc12 |
| AK043745 | 1.871 | 0.0485 | Tmem71 |
| NM_027927 | 1.871 | 0.0367 | Ints12 |
| ENSMUST00000027172 | 1.869 | 0.00788 | Ica1l |
| NM_013935 | 1.868 | 0.0402 | Ptpla |
| NM_001099633 | 1.867 | 0.0129 | Dnahc9 |
| NM_023119 | 1.866 | 0.0156 | Eno1 |
| NM_025588 | 1.86 | 0.00208 | Exoc2 |
| NM_025968 | 1.85 | 0.0479 | Ltb4dh |
| ENSMUST00000090429 | 1.85 | 0.0464 | Cd59b |
| NM_011920 | 1.848 | 0.0222 | Abcg2 |
| NM_172496 | 1.845 | 0.0189 | Cobl |
| NM_175563 | 1.835 | 0.0203 | Prr11 |
| NM_025949 | 1.832 | 0.0462 | Rps6ka6 |
| XM_143339 | 1.83 | 0.00888 | Wdr49 |
| NM_026013 | 1.83 | 0.000681 | Tmem77 |
| NM_008597 | 1.825 | 0.0482 | Mgp |
| NM_001110222 | 1.825 | 0.0294 | Dcx |
| NM_175116 | 1.823 | 0.0114 | P2ry5 |
| NM_028696 | 1.823 | 0.00171 | Obfc2a |
| NM_010360 | 1.821 | 0.00192 | Gstm5 |
| NM_033561 | 1.82 | 0.00615 | Eif4h |
| NM_177167 | 1.817 | 0.00828 | Ppm1e |
| NM_001081477 | 1.808 | 0.0164 | Brwd3 |
| NM_001029912 | 1.807 | 0.0359 | Zswim5 |
| NM_183389 | 1.805 | 0.0168 | Duxbl |
| NM_183389 | 1.805 | 0.0168 | Duxbl |
| NM_019724 | 1.804 | 0.00317 | Mmp16 |
| NM_027401 | 1.801 | 0.00284 | 1700010C24Rik |
| NM_009846 | 1.795 | 0.000545 | Cd24a |
| NM_026268 | 1.794 | 0.0408 | Dusp6 |
| NM_009133 | 1.791 | 0.00372 | Stmn3 |
| NM_009384 | 1.788 | 0.00374 | Tiam1 |
| NM_181577 | 1.783 | 0.0181 | Ccdc85a |
| ENSMUST00000029259 | 1.782 | 0.0127 | Mccc1 |
| NM_023119 | 1.779 | 0.0094 | Eno1 |
| NM_172685 | 1.777 | 0.00177 | Slc25a24 |
| NM_178674 | 1.774 | 0.0337 | Fbxl21 |
| NM_023233 | 1.773 | 0.0325 | Trim13 |
| ENSMUST00000032781 | 1.773 | 0.00278 | Nox4 |
| ENSMUST00000029453 | 1.769 | 0.00302 | Vangl1 |
| NM_177052 | 1.766 | 0.00394 | Kif6 |
| NM_022032 | 1.764 | 0.0172 | Perp |
| NM_025866 | 1.761 | 0.0344 | Cdca7 |
| NM_023119 | 1.76 | 0.00799 | Eno1 |
| NM_001085378 | 1.758 | 0.0148 | Myh7b |
| NM_028031 | 1.754 | 0.0147 | Zdhhc13 |
| NM_001077202 | 1.753 | 0.00507 | Hs6st2 |
| ENSMUST00000062684 | 1.752 | 0.00714 | Tmem64 |
| ENSMUST00000027606 | 1.75 | 0.00637 | Rgs2 |
| BC100412 | 1.749 | 0.00156 | 1700001E04Rik |
| ENSMUST00000021634 | 1.744 | 0.0273 | Akr1c13 |
| NM_011627 | 1.744 | 0.00322 | Tpbg |
| NM_197990 | 1.743 | 0.0362 | 1700025G04Rik |
| NM_019413 | 1.741 | 0.0289 | Robo1 |
| ENSMUST00000021564 | 1.735 | 0.0239 | Smoc1 |
| NM_001081316 | 1.731 | 0.0195 | Dsel |
| NM_008591 | 1.731 | 0.0176 | Met |
| NM_172597 | 1.728 | 0.0426 | Txndc16 |
| NM_144860 | 1.717 | 0.00151 | Mib1 |
| NM_001080995 | 1.716 | 0.00122 | 4632434I11Rik |
| BC069874 | 1.715 | 0.025 | 2810408A11Rik |
| AK143414 | 1.712 | 0.00495 | Zfp7 |
| NM_030677 | 1.711 | 0.0308 | Gpx2 |
| NM_030709 | 1.709 | 0.0253 | Tmprss5 |
| BC085192 | 1.709 | 0.0214 | L3mbtl3 |
| NM_007483 | 1.706 | 0.00011 | Rhob |
| BC047214 | 1.705 | 0.00949 | Fbxl5 |
| NM_009776 | 1.703 | 0.0147 | Serping1 |
| ENSMUST00000102777 | 1.697 | 0.0207 | Lepr |
| ENSMUST00000024724 | 1.696 | 0.028 | Crisp2 |
| BC100412 | 1.696 | 0.00573 | 1700001E04Rik |
| NM_178705 | 1.694 | 0.0395 | Luzp2 |
| NM_178628 | 1.685 | 0.0444 | Spg3a |
| NM_172742 | 1.685 | 0.0322 | Mtmr10 |
| NM_201600 | 1.684 | 0.0405 | Myo5b |
| NM_028724 | 1.683 | 0.00308 | Rin2 |
| NM_207229 | 1.679 | 0.0364 | Plac9 |
| NM_207229 | 1.679 | 0.0364 | Plac9 |
| NM_176968 | 1.676 | 0.0252 | Nt5dc1 |
| NM_007630 | 1.673 | 0.0236 | Ccnb2 |
| AJ306625 | 1.673 | 0.00719 | Synpo2 |
| BC026813 | 1.672 | 0.0329 | 6330416L07Rik |
| NM_176860 | 1.672 | 0.0255 | Ubash3b |
| NM_013755 | 1.67 | 0.0381 | Gyg |
| NM_008829 | 1.669 | 0.0441 | Pgr |
| NM_178797 | 1.668 | 0.015 | Mlstd1 |
| XM_884776 | 1.668 | 0.00802 | EG241989 |
| BC027537 | 1.666 | 0.00233 | Gins1 |
| NM_178920 | 1.665 | 0.00906 | Mal2 |
| NM_178712 | 1.664 | 0.0209 | Gpr64 |
| NM_134130 | 1.662 | 0.0449 | Abhd3 |
| NM_001033322 | 1.661 | 0.0454 | Gucy1a2 |
| NM_008252 | 1.659 | 0.0297 | Hmgb2 |
| NM_182999 | 1.658 | 0.0398 | Rnf20 |
| NM_009704 | 1.658 | 0.0227 | Areg |
| NM_016710 | 1.658 | 0.00314 | Nsbp1 |
| NM_021470 | 1.657 | 0.0255 | Rnf32 |
| NM_027759 | 1.656 | 0.0142 | Fsip1 |
| NM_010305 | 1.654 | 0.0321 | Gnai1 |
| NM_001033214 | 1.653 | 0.0146 | E330034G19Rik |
| ENSMUST00000042842 | 1.652 | 0.0172 | Cdon |
| NM_009819 | 1.652 | 0.000555 | Ctnna2 |
| XM_983501 | 1.65 | 0.0132 | EG666383 |
| NM_183187 | 1.647 | 0.0159 | BC055107 |
| AF401531 | 1.646 | 0.0114 | Cklf |
| NM_001007460 | 1.645 | 0.00141 | Zdhhc23 |
| NM_178202 | 1.642 | 0.0205 | Hist1h2bp |
| NM_019878 | 1.642 | 0.00306 | Sult1b1 |
| NM_025284 | 1.639 | 0.00224 | Tmsb10 |
| NM_207229 | 1.636 | 0.041 | Plac9 |
| NM_008288 | 1.635 | 0.0246 | Hsd11b1 |
| ENSMUST00000032701 | 1.634 | 0.0303 | Tdrd12 |
| NM_008413 | 1.634 | 0.00444 | Jak2 |
| ENSMUST00000058159 | 1.633 | 0.00999 | Cnrip1 |
| NM_009466 | 1.632 | 0.00812 | Ugdh |
| NM_175937 | 1.631 | 0.00554 | Cpeb2 |
| NM_153162 | 1.631 | 0.00281 | Txnrd3 |
| NM_028841 | 1.629 | 0.000456 | Tspan17 |
| NM_001081208 | 1.628 | 0.0228 | Hs3st5 |
| NM_001081189 | 1.628 | 0.00101 | Uprt |
| NM_022332 | 1.626 | 0.0291 | St7 |
| NM_007709 | 1.626 | 0.00164 | Cited1 |
| NM_008252 | 1.625 | 0.0352 | Hmgb2 |
| NM_172689 | 1.624 | 0.0123 | Ddx58 |
| NM_016669 | 1.615 | 0.0137 | Crym |
| NM_008646 | 1.613 | 0.0126 | Mug2 |
| NM_001008499 | 1.609 | 0.0149 | Taar4 |
| ENSMUST00000041780 | 1.609 | 0.00143 | Endod1 |
| NM_010930 | 1.607 | 0.0351 | Nov |
| NM_001080818 | 1.607 | 0.0321 | Cdc14a |
| NM_019635 | 1.605 | 0.00531 | Stk3 |
| NM_007874 | 1.604 | 0.000857 | Reep5 |
| NM_011326 | 1.603 | 0.0153 | Scnn1g |
| NM_019985 | 1.603 | 0.015 | Clec1b |
| NM_028804 | 1.603 | 0.003 | Ccdc3 |
| NM_178202 | 1.601 | 0.0238 | Hist1h2bp |
| NM_008252 | 1.599 | 0.0432 | Hmgb2 |
| ENSMUST00000051091 | 1.599 | 0.0119 | Hist1h2be |
| NM_152915 | 1.598 | 0.0378 | Dner |
| NM_007399 | 1.597 | 0.0166 | Adam10 |
| NM_025284 | 1.597 | 0.00608 | Tmsb10 |
| NM_009817 | 1.596 | 0.0219 | Cast |
| NM_028615 | 1.596 | 0.0075 | Dppa2 |
| AF461091 | 1.595 | 0.000172 | Cspg5 |
| NM_024255 | 1.594 | 0.0294 | Hsdl2 |
| ENSMUST00000029964 | 1.593 | 0.0348 | Epha7 |
| NM_130447 | 1.593 | 0.0273 | Dusp16 |
| BC100412 | 1.591 | 0.00196 | 1700001E04Rik |
| NM_009848 | 1.59 | 0.0246 | Entpd1 |
| NM_153594 | 1.589 | 0.00421 | Pcmtd2 |
| NM_007639 | 1.587 | 0.029 | Cd1d1 |
| NM_011441 | 1.585 | 0.00441 | Sox17 |
| NM_052976 | 1.583 | 0.0362 | Ophn1 |
| NM_178202 | 1.581 | 0.0184 | Hist1h2bp |
| NM_153803 | 0.645 | 0.0151 | BC038479 |
| NM_053247 | 0.645 | 0.00795 | Lyve1 |
| XR_034456 | 0.645 | 0.00447 | LOC668888 |
| NM_022331 | 0.645 | 0.0024 | Herpud1 |
| NM_025999 | 0.645 | 0.000198 | Rnf141 |
| NM_013559 | 0.643 | 0.02 | Hsp110 |
| NM_010800 | 0.642 | 0.0197 | Bhlhb8 |
| NM_025790 | 0.642 | 0.000105 | Them2 |
| BC022225 | 0.641 | 0.039 | Kif12 |
| NM_145981 | 0.641 | 0.0197 | Phyhip |
| NM_145131 | 0.641 | 0.0182 | Pitrm1 |
| NM_031999 | 0.641 | 0.000439 | Gpr137b |
| NM_172927 | 0.64 | 0.0205 | E330026B02Rik |
| NM_023913 | 0.639 | 0.0142 | Ern1 |
| NM_180678 | 0.639 | 0.00675 | Gars |
| NM_029851 | 0.638 | 0.00786 | Dync2h1 |
| NM_007499 | 0.637 | 0.0227 | Atm |
| NM_010276 | 0.637 | 0.0198 | Gem |
| NM_008102 | 0.637 | 0.00209 | Gch1 |
| NM_019733 | 0.636 | 0.000391 | Rbpms |
| NM_030098 | 0.635 | 0.01 | Rnase6 |
| NM_031169 | 0.633 | 0.0215 | Kcnmb1 |
| NM_001011794 | 0.633 | 0.00924 | Olfr1322 |
| NM_177708 | 0.632 | 0.0102 | Rtn4rl1 |
| NM_207228 | 0.632 | 0.00658 | Tsga10 |
| NM_199449 | 0.631 | 0.00118 | Zhx2 |
| U01841 | 0.631 | 0.000811 | Pparg |
| NM_001007583 | 0.63 | 0.0175 | Best3 |
| NM_030565 | 0.63 | 0.00391 | BC004044 |
| NM_009767 | 0.629 | 0.00581 | Chic1 |
| NM_133769 | 0.628 | 5.97E-05 | Cyfip2 |
| NM_009903 | 0.627 | 0.0373 | Cldn4 |
| NM_001004173 | 0.627 | 0.00587 | Sgpp2 |
| NM_001081347 | 0.626 | 0.041 | Rhobtb1 |
| NM_010612 | 0.626 | 0.00504 | Kdr |
| NM_008018 | 0.625 | 0.0101 | Sh3pxd2a |
| NM_018852 | 0.624 | 0.0253 | Scn9a |
| ENSMUST00000022176 | 0.623 | 0.0271 | Hmgcr |
| NM_145491 | 0.623 | 0.0175 | Rhoq |
| NM_173745 | 0.623 | 0.000983 | Dusp18 |
| NM_001039934 | 0.622 | 0.000897 | Mtap2 |
| ENSMUST00000102698 | 0.62 | 0.000145 | Rapgef4 |
| NM_177769 | 0.619 | 0.0276 | Elmod1 |
| NM_009665 | 0.619 | 0.0076 | Amd1 |
| NM_019802 | 0.619 | 0.00132 | Ggcx |
| NM_009665 | 0.618 | 0.0162 | Amd1 |
| NM_011957 | 0.618 | 0.0139 | Creb3l1 |
| NM_018882 | 0.617 | 0.00504 | Gpr56 |
| NM_011406 | 0.615 | 0.0377 | Slc8a1 |
| NM_172537 | 0.615 | 0.00204 | Sema6d |
| NM_026053 | 0.614 | 0.0154 | Gemin6 |
| NM_007444 | 0.614 | 0.0149 | Amd2 |
| AK122385 | 0.614 | 0.0117 | Rab3gap2 |
| NM_029787 | 0.613 | 0.00196 | Cyb5r3 |
| NM_001099323 | 0.611 | 0.00184 | RP23-211P15.2 |
| NM_013682 | 0.61 | 0.0164 | T2 |
| NM_011311 | 0.608 | 0.0494 | S100a4 |
| NM_173028 | 0.607 | 0.000867 | Vps13a |
| NM_007674 | 0.606 | 0.0172 | Cdx4 |
| NM_148922 | 0.606 | 0.00211 | Mdm1 |
| NM_015737 | 0.605 | 0.0142 | Galnt4 |
| NM_026004 | 0.605 | 0.0011 | Nt5c3 |
| NM_023503 | 0.604 | 0.012 | Ing2 |
| NM_010290 | 0.604 | 0.00494 | Gjd2 |
| NM_198419 | 0.602 | 0.0214 | Phactr1 |
| NM_001033316 | 0.602 | 0.0203 | Ffar3 |
| NM_013885 | 0.602 | 0.00618 | Clic4 |
| ENSMUST00000026475 | 0.602 | 0.00217 | Ddit3 |
| ENSMUST00000028780 | 0.6 | 0.0122 | Chac1 |
| NM_021331 | 0.599 | 0.0213 | G6pc2 |
| NM_007444 | 0.599 | 0.0154 | Amd2 |
| NM_009218 | 0.599 | 0.00573 | Sstr3 |
| NM_177606 | 0.598 | 0.0349 | Plekhh2 |
| ENSMUST00000059595 | 0.597 | 0.0176 | Prkca |
| NM_130878 | 0.597 | 0.00252 | Pcdh21 |
| NM_008212 | 0.597 | 0.00111 | Hadh |
| ENSMUST00000031069 | 0.596 | 0.00495 | Sepsecs |
| NM_181751 | 0.595 | 0.026 | Gpr119 |
| NM_008855 | 0.595 | 0.0098 | Prkcb1 |
| ENSMUST00000046188 | 0.595 | 0.000207 | EG328644 |
| AK047968 | 0.594 | 0.0117 | Zdhhc20 |
| D82072 | 0.592 | 0.00528 | Ptgds2 |
| ENSMUST00000020497 | 0.59 | 0.00386 | Aldh1l2 |
| NM_175272 | 0.587 | 0.00744 | Nav2 |
| AK043588 | 0.586 | 0.0122 | BC026590 |
| NM_011864 | 0.586 | 0.0029 | Papss2 |
| ENSMUST00000033161 | 0.586 | 0.00257 | Scnn1b |
| NM_033525 | 0.584 | 0.0151 | Npnt |
| ENSMUST00000102925 | 0.584 | 0.00537 | Uap1l1 |
| ENSMUST00000045557 | 0.584 | 4.64E-05 | Slc7a5 |
| ENSMUST00000052700 | 0.581 | 0.039 | Ffar1 |
| ENSMUST00000001713 | 0.581 | 0.012 | Gstt1 |
| NM_026053 | 0.576 | 0.00658 | Gemin6 |
| NM_175007 | 0.575 | 0.0303 | Amph |
| ENSMUST00000102568 | 0.575 | 0.0156 | Phactr4 |
| NM_028247 | 0.574 | 0.00187 | Slc16a10 |
| NM_133995 | 0.573 | 0.0401 | Upb1 |
| NM_022030 | 0.573 | 0.0315 | Sv2a |
| NM_019516 | 0.573 | 0.0026 | Lgals12 |
| NM_008638 | 0.572 | 0.0005 | Mthfd2 |
| NM_027398 | 0.57 | 0.0101 | Kcnip1 |
| NM_018878 | 0.567 | 0.000501 | Paxip1 |
| ENSMUST00000021443 | 0.566 | 0.00814 | Mthfd1 |
| NM_015781 | 0.564 | 0.00738 | Nap1l1 |
| NM_010700 | 0.563 | 0.00442 | Ldlr |
| NM_178114 | 0.562 | 0.0094 | Amigo2 |
| NM_001080780 | 0.559 | 0.0115 | Ret |
| NM_025436 | 0.558 | 0.0112 | Sc4mol |
| XM_147850 | 0.557 | 0.0224 | BC030046 |
| AK009333 | 0.555 | 0.00714 | 2310014D11Rik |
| NM_026950 | 0.553 | 0.0137 | Ociad2 |
| ENSMUST00000112701 | 0.552 | 0.028 | Cdh7 |
| AY566864 | 0.552 | 0.0158 | A1cf |
| XM_001475233 | 0.55 | 0.00495 | 4932438A13Rik |
| ENSMUST00000069011 | 0.55 | 0.000204 | Ang |
| NM_153598 | 0.549 | 0.00256 | Ugt2b34 |
| NM_001033175 | 0.545 | 0.000454 | Cln6 |
| ENSMUST00000033201 | 0.542 | 0.00621 | Anks4b |
| NM_001081388 | 0.535 | 0.00255 | Rimbp2 |
| NM_011995 | 0.535 | 0.00079 | Pclo |
| XM_147850 | 0.531 | 0.00446 | BC030046 |
| NM_001081243 | 0.53 | 0.000405 | Filip1 |
| NM_133882 | 0.524 | 0.00763 | C8b |
| ENSMUST00000032198 | 0.523 | 0.0197 | Usp18 |
| NM_001081205 | 0.523 | 0.0047 | Npal1 |
| NM_009377 | 0.522 | 0.00237 | Th |
| NM_007514 | 0.521 | 0.0416 | Slc7a2 |
| NM_011299 | 0.521 | 0.00139 | Rps6ka2 |
| NM_001081262 | 0.517 | 0.0227 | 4932431H17Rik |
| NR_002900 | 0.507 | 0.0153 | Snora69 |
| NM_145399 | 0.506 | 0.00214 | Scgn |
| NM_153420 | 0.505 | 0.00391 | Acpl2 |
| ENSMUST00000025866 | 0.505 | 0.00356 | Vldlr |
| NM_026185 | 0.501 | 0.00619 | 1300007F04Rik |
| NM_001012723 | 0.5 | 0.0237 | Wfdc16 |
| NM_001005423 | 0.498 | 0.0074 | Mreg |
| NM_031197 | 0.498 | 0.0038 | Slc2a2 |
| NM_001003913 | 0.497 | 0.00231 | Mars |
| NM_021332 | 0.497 | 0.000388 | Glp1r |
| XM_911780 | 0.496 | 0.0323 | EG626952 |
| NM_001081014 | 0.491 | 0.000929 | Dennd4c |
| NM_031250 | 0.49 | 0.00179 | Ucn3 |
| NM_016717 | 0.489 | 0.0236 | Scly |
| NM_011374 | 0.482 | 0.0158 | St8sia1 |
| XM_147850 | 0.476 | 0.00707 | BC030046 |
| NM_021883 | 0.475 | 0.0394 | Tmod1 |
| NM_001080815 | 0.475 | 0.000876 | Gipr |
| ENSMUST00000058126 | 0.473 | 0.0174 | Nr1h4 |
| NM_022312 | 0.472 | 0.0046 | Tnr |
| NM_011843 | 0.472 | 0.00255 | Mbc2 |
| NM_018869 | 0.468 | 0.00426 | Grk5 |
| ENSMUST00000044231 | 0.467 | 0.00773 | Serpina10 |
| NM_183168 | 0.456 | 0.00771 | P2ry6 |
| NM_016863 | 0.451 | 0.000185 | Fkbp1b |
| NM_020279 | 0.445 | 0.00234 | Ccl28 |
| ENSMUST00000060304 | 0.443 | 0.00332 | Tox3 |
| NM_001081346 | 0.438 | 0.0305 | Rtkn2 |
| NM_021455 | 0.438 | 0.00587 | Mlxipl |
| NM_146214 | 0.432 | 0.00642 | Tat |
| NM_015744 | 0.426 | 0.00227 | Enpp2 |
| ENSMUST00000049389 | 0.418 | 0.0035 | Zdhhc2 |
| NM_018857 | 0.414 | 0.039 | Msln |
| ENSMUST00000072299 | 0.399 | 0.00873 | Vsnl1 |
| NM_019775 | 0.392 | 0.0264 | Cpb2 |
| NM_011832 | 0.363 | 0.00231 | Insrr |
| NM_008509 | 0.322 | 0.00291 | Lpl |
| NM_021391 | 0.322 | 0.000233 | Ppp1r1a |
| NM_026853 | 0.319 | 0.0122 | Asb11 |
| NM_026935 | 0.285 | 0.017 | Sult1c2 |
| NM_007843 | 0.226 | 0.0141 | Defb1 |

**Table S2. Transcripts that were changed in Sox17-GOF islets.**

List of transcripts change by >1.55 fold in Sox17-GOF islets. Red highlighted genes were upregulated, blue highlighted genes were downregulated.

**Table S3: Gene Ontology analysis of Biological Process that are significantly affected in Sox17-GOF islets.**

**Pathways and processes associated with upregulated genes:**

| **Category** | **Gene Ontology (GO) ID** | **Name** |
| --- | --- | --- |
| GO: Biological Process | GO:0048468 | cell development |
|  | GO:0007409 | axonogenesis |
|  | GO:0051239 | Regulation of multicellular organismal process |
|  | GO:0000904 | cell morphogenesis involved in differentiation |
|  | GO:0048812 | neuron projection morphogenesis |
|  | GO:0048667 | cell morphogenesis involved in neuron differentiation |
|  | GO:0008038 | neuron recognition |
| **Pathways and processes associate with downregulated genes:** |  |  |
| **Category** | **Gene Ontology (GO) ID** | **Name** |
| GO: Biological Process | GO:0002790 | peptide secretion |
|  | GO:0030073 | insulin secretion |
|  | GO:0030072 | peptide hormone secretion |
|  | GO:0015833 | peptide transport |
|  | GO:0046879 | hormone secretion |
|  | GO:0009914 | hormone transport |
|  | GO:0002791 | regulation of peptide secretion |
|  | GO:0090087 | regulation of peptide transport |
|  | GO:0090276 | regulation of peptide hormone secretion |
|  | GO:0003001 | generation of a signal involved in cell-cell signaling |
|  | GO:0023061 | signal release |
|  | GO:0046883 | regulation of hormone secretion |
|  | GO:0050796 | regulation of insulin secretion |
|  | GO:0032879 | regulation of localization |
|  | GO:0010817 | regulation of hormone levels |
|  | GO:0051049 | regulation of transport |
|  | GO:0032940 | secretion by cell |
|  | GO:0010646 | regulation of cell communication |
|  | GO:0009749 | response to glucose stimulus |
|  | GO:0060341 | regulation of cellular localization |
|  | GO:0046903 | secretion |
|  | GO:0009746 | response to hexose stimulus |
|  | GO:0034284 | response to monosaccharide stimulus |
|  | GO:0051046 | regulation of secretion |
|  | GO:0006399 | tRNA metabolic process |
|  | GO:0006082 | organic acid metabolic process |
|  | GO:0033500 | carbohydrate homeostasis |
|  | GO:0042593 | glucose homeostasis |
|  | GO:0009743 | response to carbohydrate stimulus |
|  | GO:0009308 | amine metabolic process |
|  | GO:0051649 | establishment of localization in cell |
|  | GO:0018193 | peptidyl-amino acid modification |
|  | GO:0032024 | positive regulation of insulin secretion |
|  | GO:0019752 | carboxylic acid metabolic process |
|  | GO:0043436 | oxoacid metabolic process |
|  | GO:0044106 | cellular amine metabolic process |
|  | GO:0006520 | cellular amino acid metabolic process |
|  | GO:0002793 | positive regulation of peptide secretion |
|  | GO:0042180 | cellular ketone metabolic process |
|  | GO:0006112 | energy reserve metabolic process |
|  | GO:0007267 | cell-cell signaling |
|  |  |  |

**Table S3. Gene ontology analysis of biological pathways and processes associated with SOX17 regulated transcripts**

List of gene ontology of the biological process that are involved in the Sox17-misregulated genes cluster in the Sox17-GOF islets microarray by 1.30 fold change and above (upregulated genes cluster in red, downregulated genes cluster in blue).

**Table S4: PCR primers for microarray genes validation.**

| **Genes** | **QPCR Primers** |
| --- | --- |
| Insulin | CTC CCA AAG GGC AAG CAG  GTG ACC AGC TAT AAT CAG AGA CCA |
| Pdx1 | CCA CCC CAG TTT ACA AGC TC  TGT AGG CAG TAC GGG TCC TC |
| Glut2 | CCGAACTGGAAGGAACTCAG  GGATTAAGCGGACAATTCCA |
| Foxo1 | TGCTGTGAAGGGACAGATTG  GAGTGGATGGTGAAGAGCGT |
| Atf4 | TTGTCCGTTACAGCAACACTG  GCAGCAGCACCAGGCTCT |
| GLP1R | GACCCTCAGCTGTGCAGAA  CCAGGTTCCTTCGTGAATGT |
| HDAC6 | GGGTCTCCAAGGAGGGAAT  CTTGCTGGTGGCCGTATTAT |
| Prkca | AACGAACTCATGGCACCTCT  CACTGCACCGACTTCATCTG |
| Pkd1 | TTCACCAGGAGCCCTATGTC  AGACGCTAGGGCCGAGTCT |
| Lpl | TGTGTCTTCAGGGGTCCTTAG  TTTGGCTCCAGAGTTTGACC |
| Defb1 | GAGCGGAGACAGAATCCTCC  TCTTTTCTCCCAGATGGAGC |
| Cpb2 | AAAACCTGGCCACTCTGAAA  AGGGCTTGCTCACAAGTCAC |
| Vilip-1 | TCACCGTTCTTGTCGAAGGT  TGAACCTCGAGGAGTTCCAG |
| Insrr | ACAGGTTCCAGCTCTGGGTT  TGACCTGCTCAGCTTCATTG |
| Rab27a | CATCCGACATGATGGAGAAC  AATCTAGCACTGCAGGGACG |
| Wfs | ACCTCAGTCTGTGCTTTGGG  GGGAAGAAACGGACAGAGC |
| Ppp1r1a | ACAAGTGTGGCAGGGGTG  CCCACGGAAGATCCAGTTTA |
| Gsta4 | CGTCCCCTGCCATTAAAGTA  GCTTCTTTCTCGAGTGCCTG |
| Mobp | TCCTTGGCCATTTTCTGACT  AATGAGAGCAAGACAAGCGG |
| Lipf | TGCACCTCCAAATGCAGATA  GGCAACCAGAGAAGCAGAAT |
| Use1 | TTCCTCATGTCCACCTCACA  TTTGCAGTCTAGGGCACGTT |
| Rtn1 | AGAAGAGCAGCAGCAGGAAG  ACTGTGTGTGGAGCAACTGG |

**Supplementary References.**

1. Kim I, Saunders TL, Morrison SJ (2007) Sox17 dependence distinguishes the transcriptional regulation of fetal from adult hematopoietic stem cells.[see comment]. Cell 130: 470-483.

2. Spence JR, Lange AW, Lin SC, Kaestner KH, Lowy AM, et al. (2009) Sox17 regulates organ lineage segregation of ventral foregut progenitor cells. Dev Cell 17: 62-74.

3. Wells JM, Esni F, Boivin GP, Aronow BJ, Stuart W, et al. (2007) Wnt/beta-catenin signaling is required for development of the exocrine pancreas. BMC Dev Biol 7: 4.

4. Stiles BL, Kuralwalla-Martinez C, Guo W, Gregorian C, Wang Y, et al. (2006) Selective deletion of Pten in pancreatic beta cells leads to increased islet mass and resistance to STZ-induced diabetes. Mol Cell Biol 26: 2772-2781.

5. Milo-Landesman D, Surana M, Berkovich I, Compagni A, Christofori G, et al. (2001) Correction of hyperglycemia in diabetic mice transplanted with reversibly immortalized pancreatic beta cells controlled by the tet-on regulatory system. Cell Transplant 10: 645-650.

6. Nir T, Melton DA, Dor Y (2007) Recovery from diabetes in mice by beta cell regeneration. J Clin Invest 117: 2553-2561.

7. Park KS, Wells JM, Zorn AM, Wert SE, Whitsett JA (2006) Sox17 influences the differentiation of respiratory epithelial cells. Dev Biol 294: 192-202.
